# Bridging from single to collective cell migration: A review of models and links to experiments

**DOI:** 10.1371/journal.pcbi.1008411

**Published:** 2020-12-10

**Authors:** Andreas Buttenschön, Leah Edelstein-Keshet

**Affiliations:** Department of Mathematics, University of British Columbia, Vancouver, Canada; New York University, UNITED STATES

## Abstract

Mathematical and computational models can assist in gaining an understanding of cell behavior at many levels of organization. Here, we review models in the literature that focus on eukaryotic cell motility at 3 size scales: intracellular signaling that regulates cell shape and movement, single cell motility, and collective cell behavior from a few cells to tissues. We survey recent literature to summarize distinct computational methods (phase-field, polygonal, Cellular Potts, and spherical cells). We discuss models that bridge between levels of organization, and describe levels of detail, both biochemical and geometric, included in the models. We also highlight links between models and experiments. We find that models that span the 3 levels are still in the minority.

## Author summary

In this review paper, we summarize the literature on computational models for cell motility, from the biochemical networks that regulate it, to the behavior of 1 and many cells. We discuss the distinct approaches used at each level, and how models can build bridges between the different size scales. We find models at many different levels of biological detail, and discuss their relative contributions to our understanding of single and collective cell behavior. Finally, we indicate how models have been linked to biological experiments in this field.

## Introduction

Over several decades, there has been great progress in our understanding of cell motility. In the 1980s and 1990s, the basic machinery of eukaryotic cell motion and the role of the actin cytoskeleton were discovered and refined. Regulation of motility by intracellular signaling networks was then deciphered in the late 1990s and through the 2000s. We continue to discover links between cell signaling and cell shape and function, in both normal and diseased cells. Recent efforts aim to link single cell behavior to collective behavior of many cells and emergent dynamics of tissues.

Though originally descriptive, cell biology has emerged as a quantitative science over the same time span. Mathematical and computational modeling have become more universally accepted, more closely integrated with experimental research, and more advanced in terms of methodology.

Here, we survey the state of the field, emphasizing bridges that span scales: from molecular signaling to multicellular hierarchies. We focus on the role of modeling and computational biology. Because the literature is vast and growing exponentially, we limit our review to several key themes and concentrate on 3 questions:

1. To what extent have models provided a way to bridge between the 3 levels of organization, from intracellular signaling, to single cell behavior, and to collective cell/tissue behavior?
2. What level of detail is appropriate in a computational or mathematical model? What kinds of models are suitable for a given situation?
3. What is the relationship between models and experiments in the current literature on the subject?

At each level, we consider these 3 questions in subsections with headings “Bridging scales,” “Levels of detail,” and “Links with experiments.” Like any other subdivision, this is to some extent arbitrary, as literature papers often span such categories.

Many excellent reviews are already available, including [1–4]. Some survey computational methods and others provide links to experiments. The focus on the above set of 3 questions is, to our knowledge, unique to the current review.

**Fig 1:**
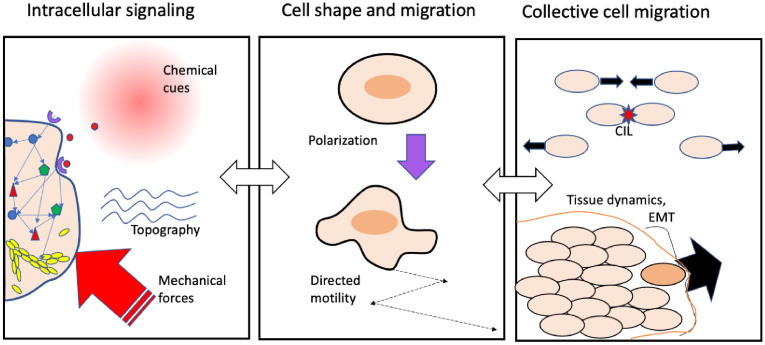
Mathematical models can be used to bridge from intracellular signaling (left), to single cell shape and motility (center), to cell-cell interactions (right). At the lowest scales, the goal is deciphering the interplay between stimuli to the cell (chemical, topographic, mechanical, etc.) and intracellular signaling networks that regulate F-actin (branched polymer) and the cytoskeleton (not drawn to scale). These interactions lead to protrusion or retraction, cell polarization, and shape changes that enable directed motility and chemotaxis. At a higher level, an aim is to link cell behavior and cell-cell interactions to the outcomes of cell collisions (e.g., CIL) and to the cohesion of tissues versus EMT, where cells break off. Interconnections exist between all layers, only 2 of which (white arrows) are shown here. CIL, Contact Inhibition of Locomotion; EMT, Epithelial Mesenchymal Transition.

**Fig 2:**
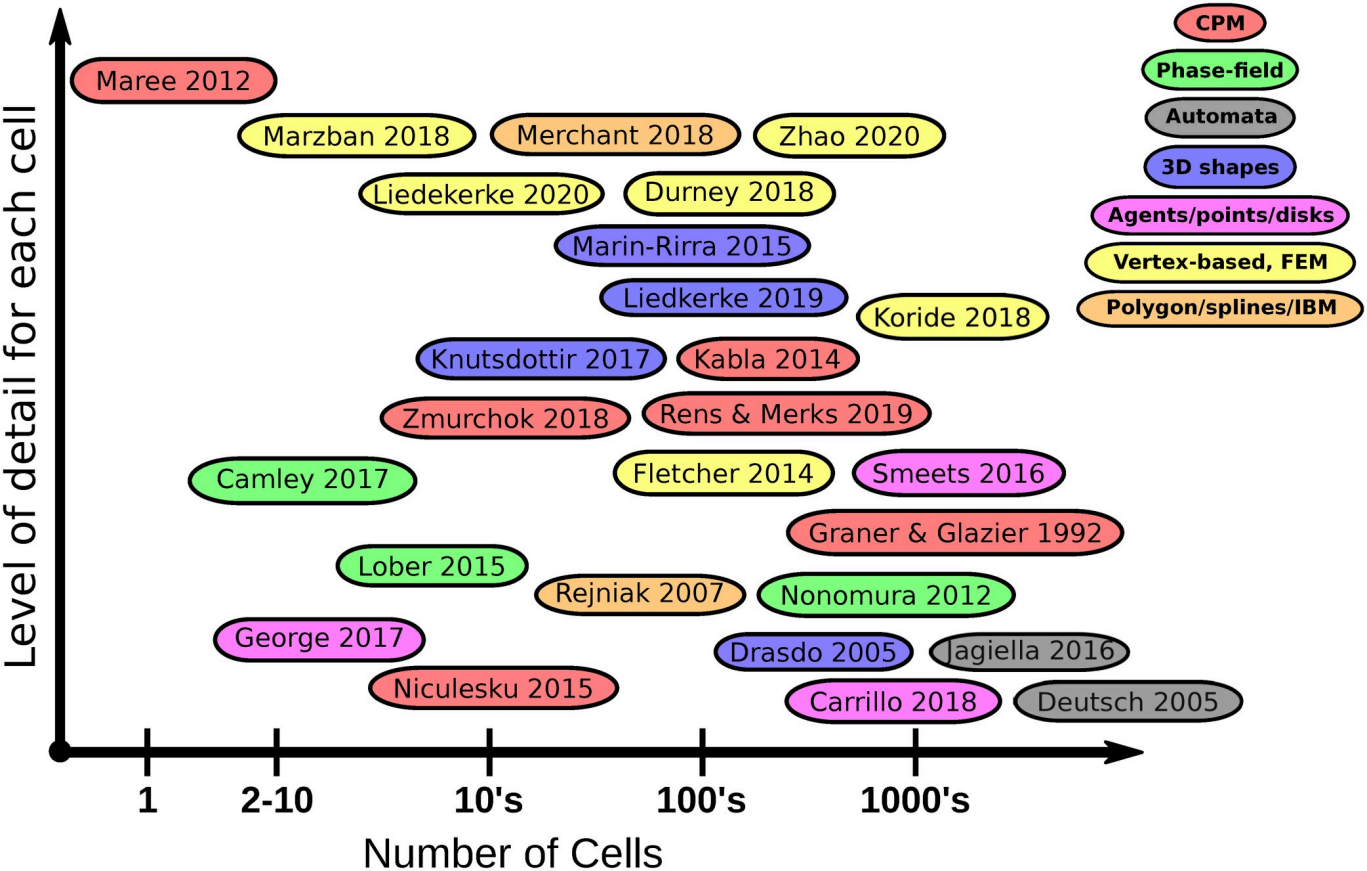
A mapping of computational models according to the number of cells (horizontal axis) and the level of detail for each cell (vertical axis). Citations of papers in the diagram (starting from the upper left to lower right: [5–30]).

The paper is organized by size-scale and level of detail. As shown in Fig 1, we start with the subcellular level of biochemical signaling (left), and move up to single cell behavior (center). We then link to small cell groups, larger groups, and tissues (right). At each level, we revisit the 3 key themes and select a few representative contributions from the literature to use as examples. A summary “mapping” of the modeling literature into levels of detail and numbers of cells is provided in Fig 2.

No one review paper can do justice to the entire field. Hence, we point the reader to related review articles that complement our own. In some cases, they cover similar ground but with distinct emphases or points of view. In [1], the authors study the issue of cell heterogeneity, its sources at various size scales, and its role in collective cell migration. They briefly discuss self-propelled particles (SPP), Cellular Potts Models (CPM), and vertex-based approaches that we also discuss in this review. They recommend further investigation of mechanical assumptions in models, and of more realistic tissue size. A thorough review of the biomechanics of collective cell migration appears in [2], with a primary focus on cancer. In contrast with other reviews, this paper also provides an excellent summary of experimental methods. Phenomena discussed include the “unjamming transition” where a cell collective changes from “solid-like” to “liquid-like” behavior and its connection to the epithelia-mesenchymal transition.

Force balance and energy-based models for single amoeboid and collective mesenchymal cell migration are compared in [3]. The authors indicate the challenge of converting between energy-based and force-based modeling platforms, and point to the significance of doing so. (See [31] for this connection in the CPM). They also describe instances of close experiment-model integration. Finally, a recent review of multiscale models in [4] has a soft-matter physics perspective and describes computational methods (CPM, phase-field, active matter, particle systems, etc.) and their physical basis.

## From cytoskeleton and intracellular signaling to cell shape and migration

How do chemical and mechanical stimuli, together with intracellular signaling shape the behavior of single cells? This question is central to the bridge between left and central panels of Fig 1.

### Actin dynamics

We briefly highlight this well-established area to illustrate examples of bridging scales, diverse levels of detail, and model–experiment synergy [32].

#### Bridging scales

Actin dynamics is studied at many levels: from association of actin monomers to form filaments [33], to biophysical force production by F-actin [34], the assembly and branching of F-actin [35], and the resultant shape and motion of cells [36, 37].

An excellent review of actin dynamics models in single cell migration, [38] provides a solid bridge between molecular scale and cell scale phenomena. The authors explain the main model formalisms and show how models can be used to understand experimental data. A review of the link from actin and its properties to cell mechanosensing and behavior is given in [39]. Reviews of the literature on actin-based cell migration reveal a mature field, where quantitative methods and theory have ripened in tandem. This synergy has benefited greatly from biochemical pioneers, such as Tom Pollard (Yale University), who helped to nurture an appreciation for mathematical and physical modeling in the community.

#### Levels of detail

The pair of papers [33, 40] aptly illustrates the dichotomy between highly detailed computational models [40] and more conceptual minimal models [33]. On one hand, the highly detailed [40] synthesizes a large body of experimental data for actin assembly and branching, including many actin-related component. At the opposite extreme, the models of [33], as well as later papers [36, 37] emphasize physical principles, universal properties, and general insights. In some sense, the overall predictions of both model types can be compared. The minimal models are more tractable for analysis, parameter sweeps, and overall insights, but are harder to connect to detailed molecular biology experiments. The complexity of highly detailed computational models (matching molecular experiments) makes it harder to navigate their results, which are more like a “1-to-1” map. Such maps may be important for those who “live in the neighborhood,” but are essentially baffling for newcomers. While distinct, one could argue that these approaches are complementary, so that overall insights can be obtained from one, and specific details from the other. The dichotomy between the detailed and the simplified models will reappear throughout this review, serving as 1 hallmark of distinct views.

#### Links with experiments

A review of the experiments and theory for actin dynamics in the motility of keratocytes is provided in [41]. A retrospective that emphasizes the importance of theory, and case-study where theory has led the way is described in [42]. Notable contributions linking experiments to theory for cell shape include [36, 37], where shapes of cells are classified and related to actin branching and treadmilling.

### Signaling networks

Fluorescence resonance energy transfer (FRET) microscopy led to breakthroughs in visualizing the activities of signaling proteins that regulate the cytoskeleton. This has enabled live imaging of spatiotemporal activity of the Rho family GTPases [43].

The roles of Rho family GTPases (Cdc42, Rac, and Rho) in regulating actin assembly and myosin contraction are well–known [44, 45]. These switch-like proteins are central regulators of cell migration, funneling signals from the cell’s environment to downstream components that shape its motility. Cdc42 and Rac promote F-actin assembly and cell edge protrusion, whereas Rho facilitates myosin light chain (MLC) phosphorylation, activating myosin-based cell contraction. Rho also promotes F-actin through formins and is a Rac antagonist. The importance of GTPase dynamics in cell motility [46] has also attracted modelers to this arena.

A review of GTPase spatio temporal models can be found in [47]. A classic paper on modeling spatio temporal dynamics of signaling in cells (not necessarily GTPases) is [48], a paper that emphasizes universal principles. We see how commonly shared basic motifs combine to form complex dynamics [48]. Many lessons learned from this approach can be applied directly to studying GTPases or other signaling networks. In general, the community stands to benefit from more expository papers of this type, where the ingredients that combine to set up specific dynamical signatures are exposed. See also [49] for a popular example of this type, based on years of experience with models of cell cycle regulatory networks.

#### Bridging scales

Several papers link the dynamics of GTPases to cytoskeletal assembly and cell shape. Among these are experimental [50, 51] and computational [5, 52] works. A review of signaling that organizes the cell front and rear in Dictyostelium discoideum is given in [53], who also survey models of these as excitable systems. Some of these papers are described in fuller detail below.

#### Levels of detail

GTPases act like molecular switches that cycle on and off the cell membrane and interact via crosstalk through effector proteins. A large collection of proteins participate in GTPase signaling: Guanine nucleotide exchange factors (GEFs) activate and GTPase-activating proteins (GAPs) inactivate the GTPases with varying degrees of specificity. GTPases are also sequestered in the cytosol by binding to guanine nucleotide dissociation inhibitors (GDIs). In some models, notably [54, 55], the details of the binding and mechanisms of activation–inactivation are lumped into phenomenological terms such as Michaelis–Menten or Hill functions. In [55], emphasis on cross-talk of Cdc42, Rac, and Rho and on spatial polarization comes at the expense of the molecular steps themselves (some of which remain unknown). In [56], more detail on such steps is modeled and eventually reduced to 3 ordinary differential equations (ODEs) using a quasi-steady-state approach. The authors focus on the role of a Cdc42-GEF in oscillatory phenotype in neuronal growth cone motility. In contrast, [57] concentrate on GDI binding of GTPases in a highly detailed computational model (originally crafted in BioNetGen and Virtual Cell).

Crosstalk between GTPases is modeled in great detail by [58], who constructed a Boolean model for epidermal growth factor (EGF) signaling to Rac and Rho. Their model has 38 intermediates and multiple reactions that activate or inhibit components. (See also [59], who combines PhysiCell with MaBoss to allow the modeling of intracellular signaling networks as Boolean networks). At lower level of detail are Rac-Rho mutually antagonistic models that leave out the forest of interacting nodes [60].

Simpler models for single GTPase include [61] for cell polarity and [62, 63] for cell shape. There, assumptions are made to condense underlying complex interactions into simpler “rules” of behavior. For example, “high GTPase activity leads to outwards protrusion force at the cell edge” (in the case of Rac) or “contraction” (in the case of Rho) [64, 65]. An advantage of this simplification is that simple models consisting of few partial differential equations (PDEs) allow for well-honed methods of applied mathematics, including nonlinear dynamics, stability theory, asymptotic analysis, and/or bifurcation theory to explore model predictions, parameter dependence, and regimes of behavior.

Serendipitously, simplified signaling models, like [61], occasionally also expose interesting mathematical structures to study. A case in point is “wave-pinning” [61, 66]: a wave of GTPase activity initiated at 1 end of a cell stalls before reaching the opposite end, resulting in robust “cell polarization.” Simplified models also permit numerical implementation in more complex geometries. For example, in [67], a wave-pinning model for a single GTPase in a single 3D cell is implemented by bulk diffusion methods. The cell does not deform, but the spatial localization of the GTPase is quantified in a fully 3D geometry. (See also [68], who considered neutrophil fluidization, and [69], who considered polarization in a 3D sphere).

At a gradually increasing scale are models that include not just GTPases (Rac, Rho, and Cdc42) but also other layers (phosphoinositides, actin, Arp2/3, and myosin) that interact to regulate cell polarity in response to stimuli [70], or dictate cell shape and directed motility [5, 52]. In the latter 2 papers, a single moving “keratocyte” is represented using a Cellular Potts Model (CPM), an energy minimization agent-based platform for computing cell shapes. It is shown that GTPase signaling can account for cell polarization, reorientation [52], and resolution of conflicting cues or obstacles [5]. (Compare with [71], who employ phase-field computational methods toward similar goals).

By way of comparison, in another more detailed approach, in [72], a cell is modeled as having a solid boundary that is moved using a Stefan condition. For the chemical signaling, Rho, Rac, 2 species of GEFs, F-actin, and G-actin are included. The model accounts for the basic repertoire of neutrophil motility.

All in all, models of GTPase signaling have taught us several valuable lessons, some with universal ramifications. First, modeling has provided clues to the functional significance of the seemly strange cycling of GTPases between membrane and cytosol: namely, the separation of diffusion rates resulting from these distinct compartments could be playing a role in pattern formation processes, an ingredient enabling GTPase localization or patterning in cells. Second, the stripped-down models have shown that chemical polarization in a cell need not depend on crosstalk between multiple types of GTPases—it can be set up by a single member of the family, given sufficient positive feedback and some depletion of its cellular pool [61]. Groups are still occasionally rediscovering on their own, the link between cell size and cell polarization that was implicit in [66], suggesting the need for more expository reviews of mathematical results. An important concept introduced in [5], but not yet fully recognized in the community, is the synergy between GTPase dynamics and its effect of cell shape and boundary curvature of the cell edge. Simply put, the “motion by curvature” of the chemical system interacts with boundary conditions to accelerate the dynamics. Altogether, the appreciation of GTPase signaling has benefited greatly from a host of distinct modeling and mathematical approaches.

#### Links with experiment

A review of the links between models of cell migration and experimental data (image processing, cell tracking, and feature extraction from 1 cell to many) is given in [73]. Here we focus more specifically on experiments that highlight intracellular signaling.

The work by [70] provides data for the reorientation and polarity responses of HeLa cells from various starting cell states (polar, anti-polar, or nonpolar). The authors showed that an internal circuit of signaling (Cdc42, Rac, Rho, and phosphoinositides) could account qualitatively for the observed responses of these cells in microfluidic channels, with an externally controllable response to Rac. In both this and the follow-up [74], the 1D geometry of the channels helps to reduce the geometric complexity of cell shape, allowing for a better match to 1D spatial model representations.

How are models for GTPase crosstalk experimentally linked to cell morphology and motility dynamics? In experimental results of [51], the authors showed that signaling circuit of mutually antagonistic Rac and Rho could affect not only the dynamics of F-actin, but also shapes and migration of mesenchymal breast cancer cells. Together with a mathematical model for the Rac-Rho interactions, they were able to manipulate the Rac-Rho competition, demonstrate bistable states, and show that manipulating the system by inhibiting PAK (a kinase that mediates inhibition of Rho by Rac) displayed hysteresis characteristic of bistable systems. The significance of this paper is that it demonstrates a direct link between a simple hypothetical model for the way that Rac and Rho GTPases operate in a cell and the next level up, that of overall cell morphology.

Sometimes, individual papers provide only part of the story, but taken as a whole, a series of papers gives a broader view. The sequence of work in [50, 60, 75, 76] explore how Rac-Rho mutual antagonism is linked to cell morphology. In [75], the authors experimentally manipulated Rac and Rho activities to show spread or contracted cells. Interestingly, they found that combining constitutively active (CA) Rac and Rho simultaneously results in mixed morphologies in human glioma cells. The fact that high Rho and high Rac activity produces a mixture of possible coexisting stable steady state cell shapes was independently predicted in a purely modeling study by [60]. Related experiments by [50] on cell shapes were also later modeled and explained in a follow-up paper by [76]. These papers demonstrate that relatively simple stripped-down depictions of cellular signaling “modules” (such as Rac-Rho) can account for important and unexpected observations at the level of the cell as a whole.

The paper [77] explores how 3D collagen microtracks and confinement affect cell migration. The authors find that the degree of cell-extracellular matrix (ECM) interactions are key determinants of speed, morphology, and cell-generate substrate strains during motility.

In recent times, a clear link has been established between GTPase activity and mechanical tension experienced by cells. A pioneering experimental paper that showed this 2-way feedback is [78]. The effects of forces on the Rho family proteins, including the involvement of GEFs that activate Rac1, RhoA, or Cdc42 are reviewed in [79]. Some GEFs respond to cyclic stretch, and others to tensile force or shear stress and substrate stiffness. Rac1 and Cdc42 are activated by stretching of adhesion bonds [80]. Rho is mainly used in maintenance of focal adhesions, but it appears to play a prominent role in cell–matrix interactions.

Strain and strain gradients affect cell orientation. Single cell experiments with cyclic stretch are described in [81]. The authors suggest that the Rho-ROCK pathway that regulates myosin light-chain activity is responsible for sensing and responding to strain gradients.

Overall, it appears that the link between mechanical stimuli and GTPase signaling is still young, providing ample opportunities for creative modeling. So far, there is no consensus on what are the “takeaways” from the models so far. Further, it would appear that this gap should be filled if we are to successfully bridge between 1 cell and many, since the mechanics of cell collisions, as well as cell collective migration, entail sensing of both chemical and mechanical stimuli in the interacting cells.

## From single to collective cell behavior

**Fig 3:**
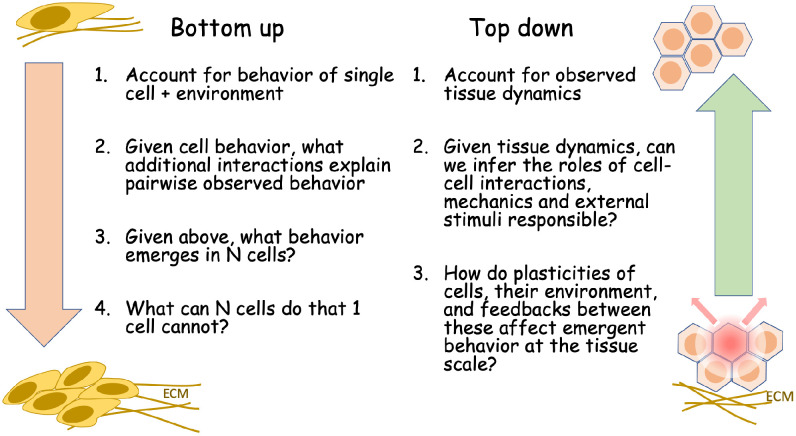
Modeling goals can be classified into broad categories that span levels of hierarchy. Some models attempt to span knowledge of single cell behavior plus interactions to predict emergent multicellular behavior (bottom up, left), whereas others start with observations of tissue dynamics and seek to infer underlying rules, feedbacks, and cell-cell (c-c) interactions that lead to those observations (top down, right).

While the meaning of “collective behavior” is intuitively clear, what is less clear is how to specify the transition between a collection of agents, acting individually, and the collective behavior of the group (right panel, Fig 3). To some extent, the same issue arises in macroscopic models for swarming animals or interacting particles. As groups grow and interactions between members increase, new distinct properties emerge at the level of the group that were absent at lower levels of organization. Characterizing, quantifying, and understanding such emergent properties remains the single most interesting and elusive goal in bridging between single and collective phenomena.

Unsurprisingly, physicists have grappled with similar questions in inanimate systems. This goes 2 ways. If we observe the dynamics of the group, can we infer underlying interactions? This is a top-down approach; see Fig 3. Conversely, given rules followed by individuals, what can we predict about the structure and dynamics of the group? For example, how do “rules” for cell–cell interactions such as coattraction, contact inhibition of locomotion (CIL) affect cell collectives? In cell biology, we add the question of how rules of behavior of cells are to be associated with specific molecular/signaling pathways inside the cells.

From physics, we gain various concepts and techniques such as order parameters, e.g., average movement direction, normalized separation distances, or migration persistence measures. Mathematicians have also contributed with useful methods, such as dynamical systems, ODEs, PDEs, and bifurcation methods. More recently, the use of topological data analysis (TDA) has also entered the mix, to help address some of these questions. The examples provided by [82–85] show how TDA can be applied even more effectively than the traditional physics-based order parameters to compare data and model output. Such novel methods could help to begin addressing questions where model hypotheses are to be compared with observed behavior.

### Small cell groups

A number of illuminating papers suggest that to understand the dynamics of tissues, we should start by first understanding 1, 2, or a few interacting cells in small groups [25, 86]. (See top right panel, Fig 3). Then, the behavior of single cells and the effect of cell–cell interaction can be explored in detail. Collisions between cell pairs or “cell trains,” contact inhibition of locomotion, and similar responses fall into this category, as do “cell swarms.” The migration of neural crest cells is 1 example of a loose cell swarm, as reviewed in [87]. Adherent cells in tissues (and their cohesion in epithelia) will be discussed in another section.

A number of key questions posed by papers in this area include the following:

1. What mechanism, chemical and/or mechanical, can account for CIL in interacting cell pairs [7]?
2. How do cells reconcile conflicting cues [74, 87]?
3. Under what conditions would a cell reverse its direction [74]?

The study of small numbers of interacting cells provides a useful paradigm for connecting molecular mechanisms to group behavior, an area that has only recently received the attention it deserves [86].

#### Bridging scales

Phase-field methods have been used to model cell shape and motility in 2 spatial dimensions (2D) [71]. The outline of a cell is represented by a level curve of some function *ϕ*(*x*, *y*, *t*). For example, in [88], a minimal model for Rac signaling was used to describe the shape and motility dynamics of a single phase-field cell. The model is then extended to pairwise and cell–train interactions in a narrow 2D strip. Interestingly, the authors had to assume an intracellular signaling “inhibitor” that is activated at cell–cell contacts to obtain appropriate behavior. This is one of the earliest models spanning the three levels of organization, from subcellular, to single and multiple cells. The model exploits a reasonably simple level of detail at each stage to reproduce several behaviors, such as reversals, cells walking-past one another, cell sticking, and forming a “cell–train.” We feel that this paper provides good prototype to be emulated by community members: a clear and well-studied intracellular system, linked to evolving cell shape, with feedback from cell–cell interactions. The paper leads to natural follow-up questions, amenable to experimental investigation: what cell components play the role of the putative “inhibitor”?

Models for neural crest cell (NCC) swarms in vivo and in vitro were proposed in [87, 89, 90], focusing on distinct behaviors of leader and follower cells. (See “Links with experiments” for a summary). For the same NCC system, the paper [7] developed a multiscale computational model linking signaling to cell group migration. A Rac-Rho GTPase circuit was represented by differential equations (ODEs) at nodes on the perimeter of a deforming polygonal cell. Co-attraction was represented as a non local tendency of cells to cluster, short range contact inhibition by up-regulating Rho at nodes in contact with other cells. Stochastic noise in Rac activity generated characteristic run lengths and reorientation seen in typical NCCs.

In [7], it was shown that these underlying biochemical systems could account for CIL. This study can be compared with the GTPase and inhibitor system described above, [91]. Coherent migration of a group of *n* cells, each of diameter *d*, was possible along a confinement corridor of width on the order of $d\sqrt{n}$. This paper, [7], has a wider corridor and larger numbers of cells than [88], but confinement serves a similar function of enhancing directed cell movement. In real embryos, such “corridors” could be defined by permissive and inhibitory regions for NCC migration, as shown experimentally in developing embryos [92]. As a comparison to the models of [7, 88], in [93] a “rule-based” approach was applied to the same problem of NCC group migration, without bridging to the biochemical circuit.

In somewhat related work, [8], 2D cells are described by viscoelastic triangular finite elements. Cells can adhere to one another using linear springs. At each cell vertex, intracellular signalling controls cell protrusion and cell–matrix adhesion. The signaling networks consists of Rac and several other components, including the downstream kinase PAK, the focal adhesion protein Paxillin, the cell adhesion molecule cadherin, and Merlin, a protein implicated in contact inhibition of cells in a feedback loop with Rac1 and PAK. Key findings of this study were (1) cells elongate on stiffer substrates; and (2) stiffer substrates allow for better directional guidance and collective cell migration. Interestingly, the authors state that the principal role of intracellular signaling is to coordinate cell movement direction over distances of ≈ 500*μm*.

#### Levels of detail

There are many “simple” SPP models in the literature on collective cell behavior, reviewed, for example, in [94, 95]. It is relatively straightforward to create rules that result in collective behaviors, but harder to do so when more details are included.

In 1 SPP cell model in a 1D spatial domain, [25], CIL and force-induced cell repolarization result in properties of collective cell behavior. Rules governing pairwise interactions and probabilities for CIL are derived from experiments by [96] described in the next section. It turns out that there is an optimal number of cells in a group for migration persistence. This paper joins [70, 74, 96] and others in proposing and using simplified 1D geometry to examine the basics of cell–cell interactions. The synergy between papers [25, 96] is very helpful, and points to the need for more paired experiment-model studies.

SPP models are easily extended to higher dimensions, and/or more realistic pairwise forces. For example, [29] considers a cell swarming model in 2D that is closely related to spherical cell models in [27, 97]. While [29] neglects cell–cell friction, the paper couples the cells’ compressibility, which plays the role of a local force of repulsion, to adhesion due to long protrusions such as filopodia that leads to nonlocality.

The highlight of [29] is the inclusion of these non local cell–cell attraction forces. In this way, the paper makes a seminal link between extended cell–cell interactions and a body of theoretical work on non local interactions in swarms or physical particles. The same paper also features the concept of H-stability (preservation of bounded density as the number of particles increases). This idea is used to ascertain correct cell spacing in swarming models, facilitating links to experimental observations. This paper hence imports a panoply of techniques, methods of analysis, and results, from theoretical work on non local models dating back to the 1990s [98], and inspires new ways of interpreting experiments.

Parallels with macroscopic swarms and flocks are useful, so long as we remember that these operate in vastly different friction regimes. (Cells experience an overdamped regime, where inertial forces are negligible). In yet another example of a cell swarming model, the paper [99] accounts for the development of stripes in zebrafish pigmentation patterns. The model comprises 2 cell types, with cell differentiation and death, and successfully reproduces a wide array of observed wild-type and mutant patterns.

In a different approach at a lower level of detail, [100] describes a group of border cells in Drosophila as a single sphere to determine the role of cluster size in chemotactic migration speed. The authors assume a spherical cluster moving by Stokes’ law in a viscous environment. The migration force is proportional to cell surface receptor occupancy in an exponential gradient of attractant. The results show good fits to both normal cells and cells that have a deficiency of receptors. The same biological problem is treated in [101], using a force-based approach for individual spherical cells (rather than cluster as a whole as in [100]) in 2D, followed by 3D (for small clusters of up to 8 cells). The computation incorporates cellular adhesion and repulsion, with some stochastic effects.

At the next level of detail, the shape of interacting cells is explicitly represented. The papers [91, 102] apply phase-field methods to model cell shapes in 2D with intracellular signaling. Interestingly, the signaling system is itself “minimal:” The authors assume basic “wave-pinning” GTPase dynamics, as in [61], with additional stochastic elements. The levels of internal GTPase give rise to either protruding or retracting forces on the cell edge. Then, [91] models the rotational cell motion when 2 cells interact (compare with [31] who showed a similar behavior in CPM cells). For coordinated movement, the directions of these gradients need to be linked. A key finding of [91] was that the emergence of rotational motion is strongly dependent on the type of cell–cell coordination mechanism (which includes the previous “inhibitor” field and other phenomenological cell polarization mechanisms).

We see gradual ascent in the numbers of cells considered and level of detail. We now find groups addressing the question “What can cells do well together that they do poorly on their own?” For example, collective chemotaxis was shown to be better in groups than in single cells [18, 103, 104]. (See also the related work by [22] for larger cell groups, reviewed in the next section).

Overall, our sampling of the literature reveals a paucity of models at the small-cell group level, relative to subcellular and tissue-scale modeling. We believe that papers such as [29] open opportunities for swarm-centered modelers to make a contribution to the cellular biology realm, by translating numerous results of physics and mathematics, suitably reinterpreted, to cells. We also contend that understanding the ways that cell interact in small groups is an important and relatively tractable step to formulating more informed tissue-scale models.

#### Links with experiments

Pairwise interactions of MTLn3 rat breast adenocarcinoma cells inside microfluidic channels with chemotactic cues were quantified in [74]. These experiments provide important information for the spatio temporal evolution of GTPase activity during cell collisions. When cells collided in [74], they either stalled, adhered and moved together, or exhibited CIL. More recently, [105] studied how mechanics, and cell polarity drive collective cell migration in larger groups of cells. More experiments of this type would be very important in laying the groundwork for understanding pairwise cell interactions and in directly informing the development of models. Such models could then be used to interpret and deepen experimental observations.

In a similar vein is [96], a study of CIL experiments on micropatterned surfaces, where data were collected for the probability of cell repolarization after contact with another cell. The authors also explored persistence of cell trains, bridging from pairs to larger groups. An agent-based model was then used to predict cell–train outcomes based on the observed repolarization probabilities.

A model that replicates the experimental setup of [106] appears in [107]. Flat cylindrical model cells are assumed to align ECM fibers. Primarily a single-cell model, it is also extended to 2 interacting cells where, interestingly, leader-follower behavior is seen: The leader creates a “track” of aligned ECM fibers that the second cell can follow.

A number of works shed light on the molecular basis of cell–cell interactions. For instance, Wnt signaling is a ubiquitous pathway that regulates polarity, migration, and many other cellular processes. Wnt signaling at cell–cell interfaces that locally upregulates Rho is studied in [108]. Ephrins form cell–cell ligand–receptor bonds that funnel signals to GTPases to control cell repulsion or attraction. Involvement of Ephrin receptors in CIL [109], effect of tension on CIL [110, 111], and on the activation of RhoA [112] have been similarly studied.

Experiments on migration of primordial progenitor cells in the developing zebrafish [113] established the importance of guidance cues such as repulsion due to ephrins. Physical barriers also guide cell migration and organ placement. Actin was visualized for cells colliding with barriers expressing ephrin receptors. A particle-based model with an attraction–repulsion Lennard-Jones potential was then used to test the effects of reflecting and nonreflecting boundaries. (The Lennard-Jones potential is composed of power functions of the form *r*^−12^ and *r*^−6^ for local repulsion and long-range attraction).

NCCs chemotax toward some cell types in a chase-and-run behavior [114]. For example, NCCs chase the placode cells, namely cells that are destined to form feathers or teeth or hair follicles. Before contact, each cell has high Rac at its front. Upon contact, the cell-adhesion protein N-cadherin, at the cell–cell interface, inhibits Rac in both cells leading to separation and escape of the placodes. Once the cells separate, chemotaxis is reestablished.

The group of Danuser [115] used high-resolution traction-force microscopy (TFM) to measure traction forces for a single cell and small groups of 2 to 6 epithelial cells. They then used a thin-plate finite element model (assuming homogeneous elasticity) to reconstruct forces along the interfaces between cells in a cluster. It was shown that the forces were correlated with E-cadherin localization. The authors reduce the cell group to a network representation, with vectors connecting cell centroids.

A recent paper explores the relationship between initial tissue size and the dynamics of tissue growth [116].

Experiments on the migration of chick neural crest cells in vivo and in vitro are described by several groups, including that of Kulesa. His experiments have been directly linked to models previously mentioned [87, 89, 90]. The group has investigated how cells reconcile mixed chemotactic signals, as in breast cancer cells in [74], and how information is shared between cells. The differences between leaders and followers is of recent interest in the group.

While papers such as [25, 117] argue eloquently for the need to carry out small-group cell experiments, we find this, too, to be a missed opportunity. Possibly, the in vitro work that this entails is viewed as less compelling than in vivo experiments, and so, less persuasive from the grantsmanship perspective. Hence, there are still relatively few experiments to map out the links between cell signaling and cell–cell collisions, between events inside the cell and the small-scale swarms that form. We recommend this area as a promising one for experiment-model synergy.

### A related example: Spacing of nuclei

While not directly related to cell migration, we nevertheless decided to briefly highlight the following example as it illustrates close synergy between model and experiment using modern methods. In [118], models complement experiments to investigate positioning of nuclei in Drosophila larvae muscle cells. The authors used data for many hundreds of nuclei and proposed a diverse set of particle-based models. Models were formulated specifically to test distinct sets of hypotheses for how nuclei interact with microtubules and molecular motors. Interestingly, machine learning was used for computational filtering, comparing large numbers of simulation outcomes to simple summary statistics such as nuclei positions. The filters are applied in stages, each stage rejecting inappropriate or inaccurate models that are inconsistent with the data.

Ideally, this process requires high-throughput screening, mandating both clear-cut summary statistics, and a large dataset. As noted by [118], the availability of data imposes a limitation on possible model complexity that could be adequately fit. Eventually, the best-fit model predicts that nuclei push against one another and against cell boundaries by sending out growing microtubules.

This example suggests a number of important questions for future model-experiment investigations. (1) How can one ascertain the optimal level of model complexity in the first place? (2) How do we extract clear identifiable, meaningful summary statistics from a large dataset? (positions of hundreds of cells) (3) What are optimal methods for massive filtering to obtain the best model? This example also illustrates a creative use of machine learning as a tool in understanding and filtering many possible mechanistic explanations. Clearly, this approach goes beyond the common machine learning application as a “black box” to sort or classify or learn rote features of cells or images.

### Collective cell behavior and tissue dynamics

Even before details of cell–cell signaling were known, theorists were formulating cell-based computational models for tissue dynamics. Garret Odell and George Oster [119] considered cell–cell pulling forces, coupled to a bistable stretch-sensing signaling module. They showed that this mechanism could account for local contractions that folds a tissue in a developmental process such as gastrulation. Their seminal idea still resonates today [16], now that signaling components are familiar to us.

Reviews of the recent biological literature on collective cell migration include [120–122]. (See also brief highlights in Table 1). A thought-provoking review of the biology, [86], focuses on the role of adhesion mechanics, bridging between force regulation in single cells and in collective cell migration. The paper gives detailed summary of the underlying molecular players, synthesizing a vast literature on the subject. The mechanical and mechanotransduction players at the molecular level are then linked to consequent cell-level properties. The paper proposes a rational program of experiments from 1 to 2 to many cells (see Fig 4 in [86]) to bridge the scales from single cells to tissues.

**Table 1:** Experimental summary: CIL, contact inhibition of locomotion; GEF, guanine nucleotide exchange factor; NCCs, neural crest cells; RDEs, reaction diffusion equations.

Main-Target	Main-Finding	Ref.
Rac opto-activation	Cell migration by photo-activation (HeLa cells)	[123]
Cdc42 opto-activation	Cdc42 activates Rac at the front, and Rho at the back of the cell (Immune cells)	[124]
Rho opto-activation	Rho at cell’s rear can control directional migration. Rho activity regulates switch between amoeboid and mesenchymal migration (macrophages)	[125]
Rho-ROCK pathway	This pathway senses and responds to strain gradients; cyclic stretching (single cells)	[81]
Chemotactic response	If gradient switches too rapidly, cells get stuck (Dictyostelium)	[126]
Cell polarization	Comparison of three RDEs, parameter fitting to data	[127]
Merlin	Merlin is a negative regulator of Rac and may also be regulated by the Rho pathway	[128]
Merlin	Key role in cell polarity, and leadership. Spatio-temporal data (cell monolayers)	[129]
Signaling at cell-cell interfaces.	Non-canonical Wnt signaling at cell-cell contacts causes an upregulation of Rho	[108]
Forces and GTPase activity	Cell-cell forces affect Rho activation	[112]
Topographic cues	Single cells have different organizations on grooved vs flat substrata (fibroblasts)	[130]
NCCs and placode cells	Intricate interplay between chemotaxis, integrins and their effect on internal GTPase activity	[114]
Ephrins	Ephrins in repolarization of cells (zebrafish development)	[113]
CIL and Ephrin receptors	Ephrin receptors affect CIL	[109]
CIL	Biphasic relationship between probability of CIL and collective migration	[96]
CIL	Tension built up during CIL	[110, 111]
CIL	Spatio-temporal GTPase patterns during cell-cell collisions and CIL events (microfluidic channels)	[70, 74]
Collective strand formation	Interesting difference between polarization of leader and followers	[131]
Small groups of epithelial cells	Forces are correlated with E-cadherin localization	[115]
Effect of forces on Rho and Rac GEFs	Some GEFs respond to cyclic stretch, others to tensile force or shear stress and substrate stiffness	[79]
Mechanical GTPase activation	Rac1, Cdc42 activated by stretching adhesion bonds. Rho maintains focal adhesions	[80]
Epithelia	“Push-pull” or “caterpillar” collective motion in narrow grooves, more complicated movement in wide channels	[132]
Epithelia	Sustained oscillations in epithelial sheets	[133]
Epithelia	Cells crawl in the direction of maximal principal stress. Leaders impose mechanical cues on followers	[134]
GTPases and GEFs	Coordinated waves of motion, cells at front react first (scratch-wound assay in human bronchial epithelial cells)	[135]

Several key questions appear in papers at this level.

(Q1) How do internal dynamics of cells influence the emergence of collective behavior? How do cells share and transfer information between one another? How and to what extent are mechanical forces transmitted over distance [136]?
(Q2) What is the role of the leading front in guiding collective migration? [132]
(Q3) Do actin cables or ECM fibers transmit long-range stresses [2, 131]? Are such mechanisms analogous to the non local sensing mechanisms that play a dominant role in models of animal swarms?
(Q4) What cellular and tissue-based mechanisms (long- and short-range cues, barriers, cell–cell adhesion, etc.) are essential for proper formation of organs during development [113, 137]?

Parallels between the polarization and directed migration of single cells and of a cell collective is highlighted in many recent works. See, for example, the recent perspective paper, [117], demonstrating parallels in the GTPase distribution, chemotaxis, and mechanosensing between the single cell level and tissue level.

As shown in Fig 5, many computational platforms are currently used to simulate multicellular migration. These are reviewed in [4, 95, 138–140]. Rigid or deformable spheres or ellipsoids (in 3D or 2D, Fig 5A1 and 5B1, respectively), polygonal “vertex-based,” or CPM cell representations are common. Software platforms such as CHASTE (Oxford University, [141–143], and Fig 5A2), CompuCell3D (U Indiana, [144], and Fig 5A3 and 5B2), or Morpheus (TU Dresden, [145]) have made it increasingly easier to simulate complex tissue dynamics without having to reinvent computational algorithms and graphics.

In our humble opinion, there is a vital need for support and sharing of standardized open-source software packages, with suitable plug-ins donated by groups utilizing those resources. First, such tools would save time, person-power, expertise, and expense of individual “kludges.” It would reduce duplication of effort—how many of us want to reinvent our own finite-element computations? Even more compellingly, such standardization can bring about much easier communication and appraisal of published models, and in-depth scrutiny of exactly what those models include. Having learned the basic steps of Morpheus, one of us (LEK) has become an enthusiastic user. See, for example, [146], where figures are linked directly to executable Morpheus .xml model files that produced them.

#### Bridging scales

Here we concentrate on bridging between models for biochemical signaling and multicellular behavior. In the next section, we will focus on bridges between 1 or a few cells to larger populations and from simple to more detailed geometry (points, spheres, and cell shapes) and dimensionality (1D, 2D, and 3D).

The modeling paper by [13] is a vertex-based computation of epithelial dynamics, where force balance of cell vertices includes an active contractile force on the cell perimeter due to myosin. ODEs track Rho activation by cell perimeter stretching and downstream myosin activation (by phosphorylation) at a given vertex. Vertex motion is a result of force balance. Friction and passive forces are derived from energy penalties for area constraint and from the energy of adhesion to neighbor edges. This aspect of the paper resembles the energy-based approach in CPM computations, e.g., in [16]. The authors quantified the effects of cell density on correlation lengths, and regimes of behavior such as streaming, contractile waves, group movement, rotation in a circular domain, presence of vortices, and non uniformity of myosin distribution.

Some similarities are shared by [13, 16]. Both are tissue-based computations, with ODEs for GTPase biochemistry, an assumption that GTPase leads to cell contraction or expansion, and feedback from cell tension back to the activation of the GTPase. Both papers find fluctuations in cell shape or size from the full feedback between signaling and contractility (Fig 2C in [13], and the comparable Fig 3B in [16]). The signaling biochemistry is assigned to vertices in [13] versus the cell interior in [16]. In [16], a single cell is first linked to a 1D chain of cells, and then to 2D CPM cells, where waves of cell contraction are observed. The levels of detail in these 2 papers are comparable, though [13] also includes predictions for myosin, unlike [16]. See also [147] for a similar approach restricted to 1D cell chains.

In the same general class, the work of [10] links cell contraction (in a vertex-based hexagonal cell with 6 spokes) to biochemical details for a circuit of proteins known to regulate myosin contractility in Drosophila dorsal closure. This model is more detailed on the specific known interactions of protease-activated receptor (PAR) proteins that form a negative feedback loop with actomyosin (Baz, Par-6, aPKC), including a set of 9 ODEs for components linked to myosin dynamics along cell edges and spokes. The authors account for several phases in the developmental process, including oscillations observed in the tissue (using time delays) and the eventual contraction of the tissue. By way of comparison, [148] has a greater level of detail of the cell shape, including more viscoelastic spokes and edges, but essentially no biochemical detail. An example of 3D vertex-based simulations with cell rearrangement and out-of-plane bending can be found in [149].

#### Levels of detail

The gene regulatory network involved in Drosophila ventral furrow formation is modeled at a fine level of molecular detail in [150] using a Boolean modeling approach. The model encapsulates interactions of transcription factors to explain 3 phenotypic attractor states, and identify missing and alternative pathways. Spatial distribution of cells and cell shape dynamics is not considered.

At the opposite extreme, internal cell signaling is omitted in favor of positions and spatiotemporal dynamics of cells. SPP “agent-based” models with forces of attraction, adhesion, and repulsion include [29], as reviewed in a previous section. There, nonlocal forces are considered in groups of hundreds of cells.

Modified agent-based models in [151] can be compared to the cell-pair study in [91]. Additional assumptions are included in [151], notably Vicsek-type alignment, cell membrane curvature, elasticity, actomyosin cable force, and a tendency to move in the outwards normal direction. The authors show the dependence of tissue wound-healing velocity on the width of a confining stripe. On a similar topic, CPM computations are used in [152] to investigate the effect of adhesive micro patterns on the motion of single cells and collective cell migration. Similarly, modified Viscek-type models are considered by [25] endowing each cell with a polarization direction. Various types of cell–cell polarity coordination mechanisms are considered.

Yet another modification of particle-based models is [153], where a cell is represented by a spring linking 2 discs, to depict the “cell body” and the “pseudopod.” The model demonstrates various outcomes of binary collisions in a 2D domain, as well as order–disorder transitions and velocity waves in large 2D cell groups with and without CIL. This paper explicitly bridges from 1 to many cells.

At the next level of complexity, we find representations of cells as spheroids or ellipsoids (Fig 5A1, [27, 154–156]). Drasdo computed a 2D growing monolayer [154], and later extended it to 3D [27]. Palsson built 3D simulations of deformable off-lattice ellipsoids, with cell division, chemotaxis, cell–cell adhesion, and volume exclusion. The platform has been used to model the dynamics of Dictyostelium aggregation and cyclic AMP (cAMP) signaling [155], as well as the posterior lateral line primordium, a cluster of about 100 migrating cells in zebrafish embryos that generate sensory structures on the surface of the fish [14].

Other recent papers add variations on these themes. For example, [157] provides a computational model for 3D off-lattice spheroid cells, but with greater level of detail for cell–cell and cell–matrix adhesion. The model is coarse grained from the biological levels of 10^6^ down to 100 sites per cell. After quantifying the stable distance between cell pairs (for a given set of forces, intrinsic cell properties, and adhesion sites), the authors go on to show the behavior of a larger group of cells.

Recently, these approaches have been extended to more finely resolve cell shapes in 3D [9]. Here, each cell is a triangulated 3D object, composed of discrete viscoelastic elements. Simulations track up to a thousand liver cells. While computationally very expensive, these approaches can provide valuable insights. For example, it is shown that highly deformable cells move more easily to heal a lesion than stiffer or more rigid cells [9]. They are also useful for specific systems where it is crucial to capture details accurately, as, for example, in drug design in silico for liver disease.

In [158, 159], a confluent epithelium in confinement is modeled by vertex-based polygonal cells, and the type of collective migration (directed flow, vortex chain, or turbulent) is related to a dimensionless quantity (“cell motility number”) that combines cell motility, cell density, and size of the confinement pattern.

In the phase-field category, the paper by [22] scales up from small numbers of cells in [91] to larger populations. Cell collisions are modeled by an energetic penalty for overlapping phase fields, and cell adhesion is described by a reaction–diffusion equation in [22]. Their model cells exhibit bistable shapes, (either symmetric or keratocyte-like), and both elastic and adherent collisions are predicted. Strong adhesion results in bands of dense closely packed cells that move as a collective. The authors compare their work with that of [160] (see Links with experiments) where a CPM model is used to describe large confluent cell rotations in a confined region. As noted by [22], the level of detail in their phase-field description is better suited for non-confluent cell models, but likely too detailed for confluent layers of cells where individual cell details average out. In [161], similar methods are extended to a 3D phase field computation for a single cell interacting with curved or grooved surface. This sequence illustrates the trade-off between geometric complexity (1D versus 2D versus 3D) and collective complexity (1 cell versus few versus many).

Overall, it emerges from many papers that the vertex-based models are good at describing epithelia, where fragmentation or EMT is absent or not important. Vertex-based models are not well suited to track cell death or fragmenting clusters, since edges and nodes are shared by neighboring cells. Breakage of a cell requires that shared edges and nodes be duplicated or reassigned, an inconvenience. To track tissue fragmentation or loss of cells, center-based models or CPM platforms have an advantage, since these represent each cell by an individual geometric object or by a set of pixels.

Continuum models are also commonly used at the level of tissue dynamics. Here, the cell identity is omitted altogether, in favor of cell densities, local flows, and material properties. Analogies are made with fluids, viscoelastic material, elastic sheets, or foams. Advantages include the ability to harness traditional methods of physics, mathematics, and fluid or engineering computational software. Examples of this approach are numerous, and we mention only a few.

One example of this class, [162], is a 3D physical description of an epithelial sheet. The authors show bistability in cell aspect ratio, bending and buckling instabilities of epithelia, and transitions that lead to formation of epithelial tubes and spheres. Experimentally testable scaling laws are given for such morphogenetic transitions. While there is no subcellular molecular detail, the connection between local cell shape and epithelial behavior is an important contribution.

In [163], the tissue is modeled as an active continuous medium, with a Maxwell viscoelastic constitutive law. A reaction–diffusion-transport equation accounts for actomyosin, whose contractility is coupled to cell motion and polarity. The authors explore predicted motion of tissue in confined regions, closure of wound gaps, and the relationship between traction forces and sizes of cohesive cell clusters. Mechanical waves, as well as tissue rigidity cycles, are observed, where fast fluidization is followed by a gradual period of stiffening.

The continuum model in [164] treats a tissue as a compressible fluid and includes both cell division and death in a type of Stefan free boundary problem. The authors show that behavior of the tissue in wound closure and in colony expansion depends on 3 parameters: 2 physical constants and the proliferation rate. They argue that all these can be estimated from limited experimental data, with examples calculated for IEC-6, a rat cancer cell line and MDCK cells, a canine kidney cell line. As the authors point out, continuum models are appropriate, provided the size of the tissue or the characteristic length of the wound is sufficiently large relative to the size of individual cells.

In some cases, an approach that combines features of both continuum and discrete cell identity is implemented. One example is [165]. Here, a 1D cell monolayer consists of a long contractile element with myosin creating a strain rate resulting in length changes. This element is flanked by cells in front and rear. Binding and unbinding of adhesion sites are also included. The model tissue exhibits durotaxis, movement directed up a stiffness gradient, under appropriate conditions on the myosin and adhesion parameters. The authors use this model to conclude that a monolayer is more effective at durotaxis than single cells.

Other examples of hybrid treatments of particle-based and continuum approaches include [166, 167]. These papers tackle the important question of how to derive appropriate continuum models from underlying SPP models. This kind of work and, in particular, expository papers that summarize the conclusions in ways that biological modelers can understand, could help link work in the literature that is currently underappreciated or not understood.

A recent work, [168], describes fingering at the front of an epithelial sheet using several of the above approaches. Cells are represented by pairs of points, as in [153], and also by Voronoi polygons for computations corresponding to experiments in [131]. The authors develop an active fluid model for the epithelium. Using these combined approaches, they demonstrate that stable fingering of the tissue edge requires leader cells. They characterize the wavenumber of the fingering instabilities (distance between stable fingers) using stability analysis of the continuum PDE model.

In summary, while the literature on tissue-scale collective migration is rapidly growing, it is still in stages of infancy as far as coherence, coordination, and clear direction are concerned. We see many exploratory steps in many individualistic directions. The list of core questions and the discovery of unifying principles is beyond the horizon, providing ample challenges for the community. We can draw an analogy between the current state of the art and the behavior of a growing but uncoordinated population of cells. There is no emerging unified front. Each is exploring independently and occasionally aggregating with a few others, but the global organizing principles are yet to emerge.

#### Links with experiments

The links between molecular players such as Rho family GTPases and epithelial morphogenesis have been known for some time. (Experimental literature reviewed in [169]). The role of Rho GTPases in the leader-follower identities and in front–rear tissue polarity is reviewed in [170].

Over the years, investigators have been asking how external constraints, geometry, strain fields, gradients, and other factors affect collective behavior. How does collective cell migration emerge from transfer of mechanical information between cells? For a cell to be a “leader,” should it be more sensitive to stimulation than other cells? Should it have elevated or more responsive GTPase activity, for example?

These and similar questions are posed in the experimental work of [134]. Here, the authors investigate the roles of the GTPases RhoA and RhoC and their GEFs in collective cell migration. It is found that cells tend to crawl in the direction of maximal principal stress, a process called plithotaxis. In their scratch-wound assay of MDCK and human bronchial epithelial cells, wounding leads to coordinated waves of motion, with cells at the front edge reacting first, followed by those successively further back. The authors speculate that mechanical cues are induced by leader cells on followers behind then by normal strain, and alongside them by shear strain to coordinate motion.

In [134], the relationship of tissue speed to distance from the leading edge is quantified using particle image velocimetry. The authors employ shRNA to knock down GTPase Cdc42 or Rac1 and many of their GEFs (screening some 81 GEFs in total). They find that the speed and directionality of the cells drop everywhere. When RhoA is depleted, there is a reduction in the spatial gradient of cell speed. They suggest that the molecular mechanism may include signaling downstream of cadherin, as well as Merlin-Rac1 signaling.

The experiments in [129] elucidate the effect of pulling stress on Rac and Rho GTPases. The authors investigated which properties of underlying molecular machinery allow for coupling between mechanical forces and correlated cell motion. The correlation length scale of collective force transmission was determined experimentally in [171]. Observed behavior was then modeled using a thin elastic sheet of height *h* with some elasticity and isotropic contraction stress. The authors investigate the emergence of leader cells and found a typical length scale of about 170 *μ*m.

A number of experimental studies have explored how epithelia behave in grooves [130, 132], various topographic surfaces, or confined settings [105], including arrays of posts [172], arrays of grooves [173], or adhesive [160] surfaces. In [132], the width of grooves and adhesive strips is varied to investigate how these affect collective migration of an epithelial sheet of MDCK cells. The authors map out the force and velocity fields in each case. Their narrowest grooves are 20 *μ*m wide, so cells are in a single file, and the authors observe “push-pull” or “caterpillar” type motion, resembling the relaxation–contraction cycles in the model by [16]. Larger tissue widths exhibit vortices of cell motion. The authors deduce that the constraints of the geometry influence cell rearrangement, as well as junctional forces between cells. (Compare with [133], who observed sustained oscillations in epithelial sheets).

Micropillar arrays form the playing field in [172] to observe collective migration and EMT in mammary epithelia and breast cancer cell lines. Dispersal of single, highly motile mesenchymal cells from a spreading epithelial front was shown to agree quantitatively with a minimal physical model of binary mixture solidification. While such a model lacks cell detail, it has several advantages, including simplicity, basic summary statistics for comparison with experiments, and availability of an analytic solution. Methods from physics can be used to inform the link between the material properties and the overall macroscopic behavior.

Both single fibroblasts and epithelial monolayers were studied in [130], showing that cells tend to be more organized on grooved versus flat substrata. Mechanical exclusion interactions, rather than strength of junctions between cells, affect the distance over which the topographic guidance signal propagates between cells. The same paper proposes a CPM computational model to describe the observations. In the CPM model, following the the style of [15], each cell is assigned a phenomenological “polarity” vector that both guides and is affected by cell displacement. Aside from the customary Hamiltonian area constraint and cell–cell adhesion energy, there is also a term for a phenomenological motile force, implemented as a “migration energy” (dot product of the polarity vector and the cell centroid position). The authors investigate how this motile force affects spatial correlation of the velocity. They observe emergence of streaming patterns. The authors also describe the influence of “leader cells,” whose polarity vector is set to an external cue. Leaders are embedded in the tissue interior, and they coordinating neighbors over some “interaction distance.”

In a similar style of experiment-model study, MDCK cells are seeded on a circular adhesive domain in [160]. Once a critical density is attained, the confluent culture collectively rotates. The same behavior is then captured in a CPM computational model that includes a motile force and a polarity term that is reinforced by cell displacement, with some persistence time. The authors show that rotation takes place when the size of the circular domain is on the order of the correlation length of cells. That correlation length, in turn, depends on the cell—cell adhesion energy, the motile force magnitude, and the polarity persistence time. Note the comparison with the purely computational paper by [22] using phase-field methods, where cell collisions, rather than adhesive confluent cells, are the subject of focus. It is also interesting to compare the CPM model in [160] with the vertex-based treatment of a similar situation in [158].

A recent paper, [105], demonstrates the fact that single cell properties affect much of the collective behavior of a cell population. In this ground breaking work, the authors link single cell Rac1 polarity to the emergent rotations of confluent cells (from few to many) confined to a ring or closed curve. A simple mechanical model captures the balance of directed motility and contact forces to demonstrate the principles at work. This paper spans subcellular to multicellular scales and provides a great example of elegant experiments to be emulated by others.

## Modeling recruited signaling networks and multiscale behavior in collective cell systems

At present, there are still relatively few models that bridge from detailed underlying molecular mechanisms through individual cell motility, all the way to tissue dynamics and collective cell migration, though the number of such papers is growing. There are 2 major issues that hamper such efforts. First, it is unclear how to deal with the problem of combinatorial complexity in trying to understand numerous players and interactions at each level. Decisions made at 1 stage affect other stages, and exploring a multiplicity of assumptions is a challenge (but see [118]). Second, complexity of the resulting models makes it challenging to determine the range of possible behavior, let alone make sense of overall principles and key components.

Few papers specifically address the signaling modules that get recruited in collective migration, beyond those that serve single cells migrating on their own. Here, we refer to signaling that is triggered by cell contact or junctions, and that specifically affects the way that cells then interact. Downstream responses might include changes in cell adhesion, migratory potential, permissive or inhibitory control of cell division or apoptosis, or relative sizes, polarity, or other aspects of cells. Input from the environment in the form of mechanical tension or topography can influence these signaling networks, promoting or inhibiting EMT. Examples of this sort include some of the following.

The role of Merlin is highlighted in [129]. Merlin, a negative regulator of Rac [128], is a member of the Ezrin-radixin-Moesin (ERM) family. When Merlin is bound to tight junctions between cells, it inhibits Rac1, but when it is in the cytoplasm, it releases that inhibition. At the same time, low Rac1 activity leads Merlin to become stabilized at tight junctions [129]. Hence, Merlin and Rac1 compete in a mutually inhibitory circuit, and a balance between mechanical and chemical factors controls Merlin activity and localization. The balance, in turn, regulates cell states that favor (high Rac, low Merlin) or inhibit (high Merlin, low Rac) protrusion of cells at the front. Spatial separation of Rac and Merlin activities can result in front–rear polarization in collectively motile cells.

Mechanical cues and ECM topography appear to influence the adhesion and migration of cells in an epithelial sheet. The signaling of YAP-Merlin-Trio (YAP, Yes-associated protein) in regulation of Rac and the expression of E-cadherin were investigated in [173] on nanostructure ridge arrays that mimic ECM. A minimal model for 2 signaling modules was proposed to account for the transition of YAP activity with distance from the front edge of the sheet. These examples of how cell–cell interactions depend on and influence both intracellular Rac gradients and adhesion motivate future modeling efforts at the multiscale level.

An intriguing stepping stone on the route to the challenging eventual targets are engineered tissues studied in synthetic biology. Since these are designed with known components, they allow for more direct development of models based on underlying mechanisms. We mention and example of this sort in what follows.

An interesting question is whether and how lessons from 1 level can be simplified into rules for components at the next level and how to best include the additional signaling pathways that get recruited at distinct levels of organization.

Here, we mention a few representative examples but recognize that others may exist of which we are as yet unaware.

### Bridging scales

In many respects, we are currently seeing first steps in models that bridge several layers of organization. Examples of this type can be found in the work of Marzban and colleagues [6, 175]. Their model combines several modules: cell polarization as in [5], a viscoelastic cytoskeleton, stress fiber structure, cell motility as in [176], and cell–substrate interaction. The authors first model the polarization, motility, and durotaxis in single cells, and then combine these with a cell–cell interaction module to simulate the rotation of tens of cells in a confining 2D annulus. The paper demonstrates a creative combination of a number of known cell representations to bridge from subcellular properties to those of the collective.

In [12], the authors study tumor spheroid growth under high mechanical compression. Their model represents cells as 3D spheres. Commonly used cell contact models (e.g., the Hertz model) do not take such large volume compression into account. The required corrections to the contact models were calibrated using a high-resolution mechanical models of cells [9]. We expect that similar approaches will in the future improve the accuracy of coarse-grained tissue level simulations.

The intersection of cellular and developmental biology has provided additional important examples of computational models that span multiple scales. Some examples predate recent efforts by 2 decades, notably computational studies of cellular slime mold, *D*. *discoideum* morphogenesis, and signaling in [177, 178]. Other examples such as [179] are purely theoretical, aimed at exploring how random gene circuits linked to cell adhesion, cell division, and some overall measure of “fitness” evolve into a zoological garden of multicellular structures. See also [180] for a recent work along a similar “EvoDevo” evolutionary developmental biology line.

### Levels of detail

By using very simple representations of subcellular events, [26] bridge from 1 cell to hundred(s) using CPM computations. The authors assumed an elementary process that mimics, but does not explicitly depict, the effect of actin on persistence of motion. Local protrusion is self-reinforcing, and decays on some time scale. First showing how 2 simple computational parameters tune the cell shape from keratocyte-like to amoeboid, they then simulate the collective migration of a monolayer. The abbreviated level of intracellular detail permits efficient computations. Note the comparison with [6], where more costly FEM computations and greater detail makes for greater computational cost and smaller number of cells that can be readily simulated. Software packages as in [181] may eventually make it more realistic to incorporate intracellular detail into multiscale models.

### Links with experiments

In creating synthetic gene networks that regulate the adhesion protein E-cadherin in real cells, Toda and colleagues [182] succeeded to design multicellular clusters that self-organize into distinct layers. The expression of E-cadherin genes was placed downstream of cell-surface notch receptors. Notch ligands on some cells activated notch receptors on neighbors, and in this way, cell–cell interactions both influenced, and were influenced by intracellular signaling. The experiments were later linked to computational models in [146, 183]. When details of the relatively “simple” genetic circuits are known, as in such synthetic biology experiments, modelers can bootstrap signaling models to learn how spatial influences and cell–cell interactions shape the emergent tissue structures.

In [184], we find a link between intracellular signaling and tissue morphogenesis. The authors show that the mutual antagonism of Rac and Rho can affect the invagination and bending of epithelial that form a lens pit in the eye development in a mouse. Rho apparently controls the apical constriction of cells via myosin, and Rac the elongation of those cells via F-actin assembly, hence accounting for the conical angle formed by each cell and the overall curvature of the tissue.

Overall, more experimental papers that probe the circuits that get recruited in the collective cell migration are needed. The papers [129, 173] on the Merlin-Rac loop and on the link to YAP and E-cadherin [173] should be followed up with more detailed computational modeling and future rounds of experiments.

## Discussion

**Fig 4:**
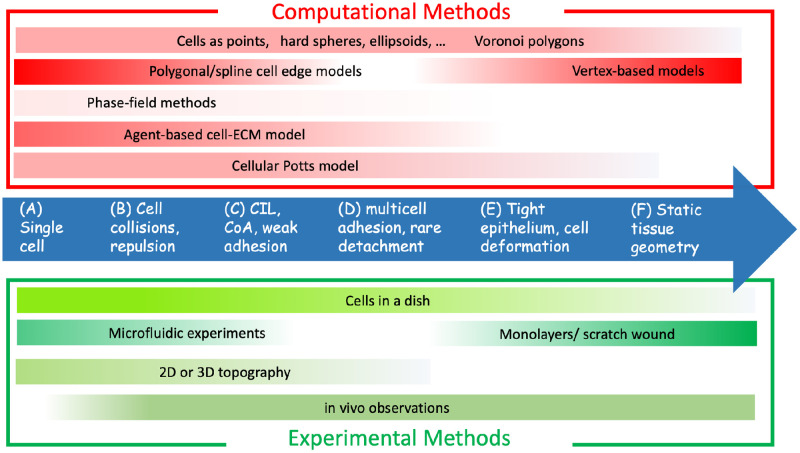
A summary of common modeling (top, red box) and experimental (bottom, green box) methods used to study cell behaviors from single cells to cell groups and up to tissues (left to right in increasing number of cells and increasing cell-cell adhesion).

In this paper, we described a small selection of modeling works on cell migration that bridge from intracellular, to cellular and multicellular scales, as shown in Fig 3. Many other excellent papers have been omitted due to space limitations. That said, even from the fraction surveyed, we find that a variety of computational and analytic methods are used at various scales. Table 2 organizes modeling papers by their subject and methodological approaches, Table 1 classifies a few experimental papers by their biological targets, and Fig 4 summarizes the ranges of relevance of both computational and experimental methods. Our review has focused on the topic of single and collective cell migration and its regulation. Likely motivated by development of disease therapies and NIH funding, or drug targets and support from pharmaceutical companies, the more medically oriented subjects such as cancer, liver toxicity, or lung morphogenesis, have fostered many generations of computational models. By comparison, the level of basic scientific computational research on multiscale cell biology modeling is still emerging.

**Table 2:** Summary of the modeling papers. Papers classified by 3 levels of organization: S, SCB, and CCB or tissue behavior. SPP models represent the cell shape statistically; common cell shape choices are spherical, ellipsoidal, or cylindrical cells. Models are categorized into: (1) CPM; (2) phase-field models; (3) vertex models; (4) particle models; (5) continuum models. CPM and phase-field models resolve cell shape well. c-c, cell-cell; CCB, collective cell behavior; CPM, Cellular Potts Models; expts., experiments; FEM, finite element methods; ODE, ordinary differential equations; RDE, reaction diffusion equation; S, signaling; SCB, single cell behavior; SPP, self-propelled particles.

Bridging scales	Levels of detail	Refs.	Experimental links
**S**	**Boolean** signaling network	[150]	Motivated by expts.
**SCB**	**3D phase-field** model	[161]	Motivated by expts.
**SCB**	**3D triangulated cells**	[9]	Links to liver regeneration expts.
**CCB**	**SPP**, pairwise forces, cell types	[29]	Pure theory, applied to expt’l design.
**CCB**	**SPP**, Spherical cells, 2D, 3D; adhesion, repulsion, random forces	[101]	Compared to live images.
**CCB**	**SPP**, 2D swarm, cell types, differentiation	[99]	Compared to fish skin patterns.
**CCB**	**vertex-based**, 3D	[149]	Integrated expt.-model
**CCB**	**polygonal cells**, 2D, 3D, viscoelastic elements	[148]	Motivated by expts.
**CCB**	Cells as **pairs of spheres**	[153, 168]	Motivated by [131].
**CCB**	**CPM** with cell polarity	[15, 130]	Motivated by expts.
**CCB**	**3D vertex based model**, epithelia	[162]	Proposes new expts.
**CCB**	**Spherical cluster**; Langevin eqn.	[100]	Integrated expt.-model
**CCB**	**Continuum compressible fluid** tissue	[164]	Reproduces expts.
**SCB**, **CCB**	**Spherical cells**, detailed adhesion dynamics	[157]	Motivated by expts.
**SCB**, **CCB**	**SPP**, disk shaped cells, ECM, Cell–ECM interaction	[107]	Reproduces expts. of [106].
**S**, **CCB**	**SPP** with alignment rules	[151]	Reproduces expts.
**S**, **CCB**	**Spherical cells**, internal details	[27, 154]	Reproduces expts.
**S**, **CCB**	**Ellipsoidal cells**	[14, 155, 156]	Motivated by expts.
**S**, **CCB**	**Vertex based**	[158, 159]	Motivated by expts.
**S**, **CCB**	**Vertex-based**; sub-cellular components	[13]	Motivated by expts.
**S**, **CCB**	**Vertex-based**, intracellular signaling	[10]	Motivated by expts.
**S**, **CCB**	**FEM**, 1D approximation of 3D	[165]	Motivated by expts.
**S**, **CCB**	**Cell agents**, repolarize after collisions	[96]	Integrated expt.-model.
**S**, **CCB**	**SPP**, leaders, followers, filopodia, chemical gradients	[87, 89, 90]	Integrated expt.-model.
**S**, **CCB**	**SPP** cells, polarity vectors, c-c coordination	[18, 103]	–
**S**, **CCB**	**FEM** cells, signaling at cell edge, adhesion	[8]	Wound-healing, compared to expts.
**S**, **CCB**	**Active media**, RDEs, actomyosin, cell motion	[163]	–
**S**, **SCB**, **CCB**	**1D or 2D cells**, ODE signaling networks	[16, 147]	Theoretical study.
**S**, **SCB**, **CCB**	**SPP**, 1D, rules for polarity coordination	[25]	Motivated by expts.
**S**, **SCB**, **CCB**	**deforming polygon** cells, signaling at nodes	[7]	Motivated by expts.
**S**, **SCB**, **CCB**	**CPM**, minimal intracellular signaling	[31]	Real cell traction forces [174].
**S**, **SCB**, **CCB**	**Phase-field** method, minimal intracellular signaling	[22, 71, 88, 91, 102]	Collision assays of [160].

**Fig 5:**
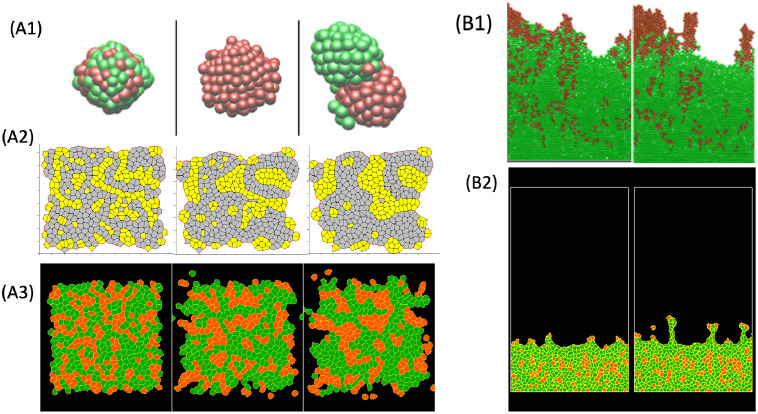
Cell sorting (left) and wound-healing (right) captured by several distinct computational methods. (A1) Cells represented by deformable ellipsoids in 3D. Simulations by Hildur Knutsdottir based on code originally created by Eirikur Palsson (A2) Vertex-based simulations using CHASTE open source platform, run by Dhananjay Bhaskar. (A3) CompuCell3D cell-sorting simulations run by Dhananjay Bhaskar. In (A1–A3), there are 2 cell types with differing adhesion strengths to self and other cell type. (B1) and (B2) show deformable ellipsoids and CPM scratch wound models with 2 cell types. Cells with weaker adhesion (red) tend to segregate to the edge of the monolayer, acting as leader cells, and causing fingering of the front. (Compare with [168]). Papers that compare distinct computational platforms include [143].

We have seen that some computational techniques that work well at the single-cell level, become too costly or excessive at the tissue level. (See also review of computational models in [4, 38, 95, 140, 143] and [185] for cancer). We also encountered topics where diverse computational techniques lead to similar predictions. (See Fig 5, and [143] for comparisons). We still find instances where 1 or another group claims that their computational method of choice outperforms others or has fewer unrealistic features. In many cases, such claims are, at best, unfortunate and overlook essential shared attributes. In other cases, they skip over relative advantages versus disadvantages of the distinct schemes. We believe that the field needs more rational comparisons of how custom-build computations perform against a host of “benchmark” test problems, as for example, shown in [143], and/or deeper comparative analysis of cell-surface mechanics models that demonstrate equivalence of distinct approaches, as in [186].

While more journals now require that simulation codes be made publicly available, in practice, this is only a half measure. Many researchers, and most biologists do not have the software packages or expertise to read, execute, and run codes in different formats. We recommend that, moving forward, the community should invest more directly in standardizing open-source software, with several specific aims: (1) ease of operation, user friendliness, and rapid learning curve; (2) basic built-in code for most major computations, including reaction–diffusion solvers, particle position and collision solvers, cell shape and cell–cell adhesion, intra and intercellular signaling, and so on; (3) ease of sharing “code,” as, for example, in the simple small Morpheus xml files—these preserve exact details of each simulation run; and (4) ease of development of new plug-ins to allow the capabilities to expand with the needs of the community. We recommend that groups invest resources in helping to develop such shared platforms and that funding bodies (NIH, NSF, and NSERC) make it a priority to support such developments. Certainly, at initial stages, this development requires teams of computational experts who can establish solid, robust platforms, and create training manuals or instructional videos to recruit new users. Morpheus, a software system developed at the Technische Universität Dresden, is 1 example along these lines, but others are needed.

Returning to questions posed in the Introduction, we can draw a few general conclusions.

### Bridging scales

Building from the bottom up, models that include detailed molecular interaction or gene networks (such as the Boolean models in [58, 150]) encode many details and occasionally reveal attractors or identify missing components [150]. They are harder to understand on their own. Detailed models such as [40] benefit from insights of preceding more basic models [33]. At lower levels of detail but still arguably “bottom up” are studies that show how certain properties of molecular components and interactions result in specific cell behavior such as polarization [5, 52, 54, 55]. Other work starts from observed cell dynamics to infer the likely signaling pathways at play [64, 70, 74] or likely underlying mechanisms [36, 118], that we could denote “top down.” The latter, [118], also demonstrates the idea that it is preferable to weed through and discuss many models, particularly some that fail, with reasons for that failure, not merely aiming for a single optimal model. This kind of process helps to build a greater intuitive understanding of the specific key mechanisms required to explain observed phenotypes.

We recommend that modelers avoid publishing large-scale models as a “fait accompli,” so as to “be the first to accurately simulate” some entire comprehensive behavior of choice. Instead, we suggest that modelers should provide due description of the steps taken in developing such models, with all informative failures and successes. This would help the community to assimilate the insights that resulted from an entire program of research.

In a related, but separate vein, the field is in need of rational consensus and standard practices for stepping between size scales and levels of organization. To draw a common analogy, our ability to easily use and appreciate global geography as well as local structure has been transformed by the way that Google maps seamlessly allows us to zoom down to street-level views and back out to the globe as a whole. The algorithms that reveal or blur over specific details as we step up or down were logically constructed for easy navigation and optimal viewing of many levels of complexity. Nothing like this currently exists in the realm of cell biology. The fact that structures and interactions change on a rapid timescale make this a tough issue, to be sure, but one deserving more attention.

Associating rule of behavior with a specific hierarchy is possible once we have sufficient familiarity with the biology and predictions of basic models. This can help to bridge hierarchies and avoid the fog of complexity. Mathematical methods such as dynamical systems, PDEs, and bifurcation analysis can help to find and account for emergent properties and universal principles in such basic models [48]. This is one of the strengths of the mathematical tools. A weakness is that these methods currently work well for small systems of differential equations, but not for large and complex systems.

Once we understand the repertoire of a single cell, we can move up to 2, 3, or many cells. Experimental observations of small cell groups provide good opportunities for understanding how to bridge from single to collective behavior. Modeling can then also explore the advantages of group migration, chemotaxis [18, 103], durotaxis [165], etc., in larger groups. For a large enough number of cells, condensing the details into simpler rules becomes expedient. For example, while polarity is represented by PDEs and patterns inside a single cell, it can then be simplified, depicted by a direction vector [15, 25] in place of a full internal gradient of Rho or Rac for multiple cells.

### Level of detail

The current computing power at our disposal allows increasingly detailed models to be constructed, with tens or even hundreds of components, and many more parameters. The temptation to create such a model and to present it as a mechanistic representation of real cells is hard to resist. Yet, before going down this very intricate route, important questions should be considered: What do we expect to learn? Might we have left out something important? Have we included superfluous detail that obscures the basic structure? Are we certain that our parameter regime faithfully corresponds with true rates and quantities? As previously noted, even though we can construct highly complex models, our comprehension of that detail is limited.

In general, best practices seem to invoke back and forth attempts to include important aspects, simplify to understand, throw out secondary factors, and go back to including detail. Simplifications lead to insights and rigorous analytic results but leave large gaps to real biology. However, returning to detailed versions of a model once the major insights are at hand (or vice versa) is helpful. While [52] explains polarization in a motile cell, for example, [61] extracts some of the key properties and interactions that guarantee that it could work in a mathematically tractable mini-model.

Furthermore, “toy models” consisting of small sets of ODEs or PDEs can help to formulate universal principles that work across many biological examples and many scales. Here, we can mention the concepts that mutual inhibition or positive feedback, which result in bistability and hysteresis. Simple “modules” consisting of specific types of interacting components, whether molecules, cells, or animals have prototypical behaviors as switches, oscillators, or other dynamics, and, with molecular diffusion or random motion, engender patterns or waves [48, 49].

### Model-experiment links

Biology is an experimental science at its core, and models that illuminate its mysteries must eventually meet and concur with biological evidence. Modern methods such as machine learning can help match a vast dataset with the best candidate model(s) as shown elegantly by [118]. At the same time, modelers, physicists, and biophysicists can make real contributions by using their craft and theories to unlock important facts that are not at all apparent otherwise [42].

Several of our examples demonstrate that some experimental papers have explicitly addressed signaling pathways that mediate cell–cell communication in collective cell behavior. As pointed out eloquently by a reviewer of this paper, “experiments can only tell [us … what are] upstream and downstream regulators. We need mathematical models to incorporate this information with the intracellular networks to form a signaling network on a multicellular level to study how a group of cells processes signals collaboratively.”

## References

[1] Blanchard GB, Fletcher AG, Schumacher LJ. The devil is in the mesoscale: mechanical and behavioural heterogeneity in collective cell movement In: Seminars in Cell & Developmental Biology. vol. 93 Elsevier; 2019 p. 46–54. 29940338 10.1016/j.semcdb.2018.06.003

[2] Spatarelu CP, Zhang H, Nguyen DT, Han X, Liu R, Guo Q, et al Biomechanics of collective cell migration in cancer progression: experimental and computational methods. ACS Biomaterials Science & Engineering. 2019;5(8):3766–3787. 10.1021/acsbiomaterials.8b01428 32953985 PMC7500334

[3] Sun M, Zaman MH. Modeling, signaling and cytoskeleton dynamics: integrated modeling-experimental frameworks in cell migration. Wiley Interdisciplinary Reviews: Systems Biology and Medicine. 2017;9(1):e1365. 10.1002/wsbm.1365 PMC5338640 27863122

[4] Alert R, Trepat X. Physical Models of Collective Cell Migration. Annual Review of Condensed Matter Physics. 2020;11:77–101. 10.1146/annurev-conmatphys-031218-013516

[5] Marée AF, Grieneisen VA, Edelstein-Keshet L. How cells integrate complex stimuli: the effect of feedback from phosphoinositides and cell shape on cell polarization and motility. PLoS computational biology. 2012;8(3):e1002402 10.1371/journal.pcbi.1002402 22396633 PMC3291540

[6] Marzban B, Ma X, Qing X, Yuan H. In silico mechanobiochemical modeling of morphogenesis in cell monolayers. bioRxiv. 2018; p. 189175.

[7] Merchant B, Edelstein-Keshet L, Feng JJ. A Rho-GTPase based model explains spontaneous collective migration of neural crest cell clusters. Developmental biology. 2018;444:S262–S273. 10.1016/j.ydbio.2018.01.013 29366821

[8] Zhao J, Manuchehrfar F, Liang J. Cell–substrate mechanics guide collective cell migration through intercellular adhesion: a dynamic finite element cellular model. Biomechanics and Modeling in Mechanobiology. 2020; p. 1–16. 10.1007/s10237-020-01308-5 PMC7990038 32108272

[9] Van Liedekerke P, Neitsch J, Johann T, Warmt E, Gonzàlez-Valverde I, Hoehme S, et al A quantitative high-resolution computational mechanics cell model for growing and regenerating tissues. Biomechanics and Modeling in Mechanobiology. 2020;19(1):189–220. 10.1007/s10237-019-01204-7 31749071 PMC7005086

[10] Durney CH, Harris TJ, Feng JJ. Dynamics of PAR proteins explain the oscillation and ratcheting mechanisms in dorsal closure. Biophysical journal. 2018;115(11):2230–2241. 10.1016/j.bpj.2018.10.014 30446158 PMC6289075

[11] Marin-Riera M, Brun-Usan M, Zimm R, Välikangas T, Salazar-Ciudad I. Computational modeling of development by epithelia, mesenchyme and their interactions: a unified model. Bioinformatics. 2015;32(2):219–225. 26342230 10.1093/bioinformatics/btv527

[12] Van Liedekerke P, Neitsch J, Johann T, Alessandri K, Nassoy P, Drasdo D. Quantitative agent-based modeling reveals mechanical stress response of growing tumor spheroids is predictable over various growth conditions and cell lines. PLOS Computational Biology. 2019;15(3):e1006273 10.1371/journal.pcbi.1006273 30849070 PMC6538187

[13] Koride S, Loza AJ, Sun SX. Epithelial vertex models with active biochemical regulation of contractility can explain organized collective cell motility. APL bioengineering. 2018;2(3):031906 10.1063/1.5023410 31069315 PMC6324211

[14] Knutsdottir H, Zmurchok C, Bhaskar D, Palsson E, Dalle Nogare D, Chitnis AB, et al Polarization and migration in the zebrafish posterior lateral line system. PLoS computational biology. 2017;13(4):e1005451 10.1371/journal.pcbi.1005451 28369079 PMC5393887

[15] Kabla AJ. Collective cell migration: leadership, invasion and segregation. Journal of The Royal Society Interface. 2012;9(77):3268–3278. 10.1098/rsif.2012.0448 PMC3481577 22832363

[16] Zmurchok C, Bhaskar D, Edelstein-Keshet L. Coupling mechanical tension and GTPase signaling to generate cell and tissue dynamics. Physical biology. 2018;15(4):046004 10.1088/1478-3975/aab1c0 29473547

[17] Rens EG, Merks RM. Cell Shape and Durotaxis Explained from Cell-Extracellular Matrix Forces and Focal Adhesion Dynamics. Iscience. 2020;23(9):101488 10.1016/j.isci.2020.101488 32896767 PMC7482025

[18] Camley BA, Zhao Y, Li B, Levine H, Rappel WJ. Crawling and turning in a minimal reaction-diffusion cell motility model: coupling cell shape and biochemistry. Physical Review E. 2017;95(1):012401 10.1103/PhysRevE.95.012401 28208438 PMC5839343

[19] Fletcher AG, Osterfield M, Baker RE, Shvartsman SY. Vertex models of epithelial morphogenesis. Biophysical journal. 2014;106(11):2291–2304. 10.1016/j.bpj.2013.11.4498 24896108 PMC4052277

[20] Smeets B, Alert R, Pešek J, Pagonabarraga I, Ramon H, Vincent R. Emergent structures and dynamics of cell colonies by contact inhibition of locomotion. Proceedings of the National Academy of Sciences. 2016;113(51):14621–14626. 10.1073/pnas.1521151113 PMC5187738 27930287

[21] Graner F, Glazier JA. Simulation of biological cell sorting using a two-dimensional extended Potts model. Physical review letters. 1992;69(13):2013 10.1103/PhysRevLett.69.2013 10046374

[22] Löber J, Ziebert F, Aranson IS. Collisions of deformable cells lead to collective migration. Scientific reports. 2015;5:9172 10.1038/srep09172 25779619 PMC4361886

[23] Rejniak KA. Modelling the development of complex tissues using individual viscoelastic cells In: Single-cell-based models in biology and medicine. Springer; 2007 p. 301–323.

[24] Nonomura M. Study on multicellular systems using a phase field model. PloS one. 2012;7(4):e33501 10.1371/journal.pone.0033501 22539943 PMC3335162

[25] George M, Bullo F, Campàs O. Connecting individual to collective cell migration. Scientific reports. 2017;7(1):1–10. 28852093 10.1038/s41598-017-10069-8 PMC5575354

[26] Niculescu I, Textor J, De Boer RJ. Crawling and gliding: a computational model for shape-driven cell migration. PLoS computational biology. 2015;11(10). 10.1371/journal.pcbi.1004280 26488304 PMC4619082

[27] Drasdo D, Höhme S. A single-cell-based model of tumor growth in vitro: monolayers and spheroids. Physical biology. 2005;2(3):133–47. 10.1088/1478-3975/2/3/001 16224119

[28] Jagiella N, Müller B, Müller M, Vignon-Clementel IE, Drasdo D. Inferring growth control mechanisms in growing multi-cellular spheroids of NSCLC cells from spatial-temporal image data. PLoS computational biology. 2016;12(2):e1004412 10.1371/journal.pcbi.1004412 26866479 PMC4750943

[29] Carrillo JA, Colombi A, Scianna M. Adhesion and volume constraints via nonlocal interactions determine cell organisation and migration profiles. Journal of theoretical biology. 2018;445:75–91. 10.1016/j.jtbi.2018.02.022 29476831

[30] Deutsch A, Dormann S, et al Cellular automaton modeling of biological pattern formation. Springer; 2005.

[31] Rens EG, Edelstein-Keshet L. From Energy to Cellular Force in the Cellular Potts Model. bioRxiv. 2019; p. 601781. 10.1371/journal.pcbi.1007459 PMC6927661 31825952

[32] Pollard TD, Blanchoin L, Mullins RD. Actin dynamics. Journal of cell science. 2001;114(1):3. 11112680 10.1242/jcs.114.1.3

[33] Mogilner A, Edelstein-Keshet L. Regulation of actin dynamics in rapidly moving cells: a quantitative analysis. Biophysical journal. 2002;83(3):1237–1258. 10.1016/S0006-3495(02)73897-6 12202352 PMC1302225

[34] Mogilner A, Oster G. Cell motility driven by actin polymerization. Biophysical journal. 1996;71(6):3030–3045. 10.1016/S0006-3495(96)79496-1 8968574 PMC1233792

[35] Grimm H, Verkhovsky A, Mogilner A, Meister JJ. Analysis of actin dynamics at the leading edge of crawling cells: implications for the shape of keratocyte lamellipodia. European Biophysics Journal. 2003;32(6):563–577. 10.1007/s00249-003-0300-4 12739072

[36] Lacayo CI, Pincus Z, VanDuijn MM, Wilson CA, Fletcher DA, Gertler FB, et al Emergence of large-scale cell morphology and movement from local actin filament growth dynamics. PLoS biology. 2007;5(9). 10.1371/journal.pbio.0050233 17760506 PMC1951782

[37] Keren K, Pincus Z, Allen GM, Barnhart EL, Marriott G, Mogilner A, et al Mechanism of shape determination in motile cells. Nature. 2008;453(7194):475–480. 10.1038/nature06952 18497816 PMC2877812

[38] Danuser G, Allard J, Mogilner A. Mathematical modeling of eukaryotic cell migration: insights beyond experiments. Annual review of cell and developmental biology. 2013;29:501–528. 10.1146/annurev-cellbio-101512-122308 PMC4148455 23909278

[39] Mak M, Kim T, Zaman MH, Kamm RD. Multiscale mechanobiology: computational models for integrating molecules to multicellular systems. Integrative Biology. 2015;7(10):1093–1108. 10.1039/c5ib00043b 26019013 PMC4677065

[40] Ditlev JA, Vacanti NM, Novak IL, Loew LM. An open model of actin dendritic nucleation. Biophysical journal. 2009;96(9):3529–3542. 10.1016/j.bpj.2009.01.037 19413959 PMC2711424

[41] Mogilner A, Barnhart EL, Keren K. Experiment, theory, and the keratocyte: An ode to a simple model for cell motility In: Seminars in cell & developmental biology. vol. 100 Elsevier; 2020 p. 143–151. 31718950 10.1016/j.semcdb.2019.10.019

[42] Pollard TD. Theory from the Oster Laboratory leaps ahead of experiment in understanding actin-based cellular motility. Biophysical journal. 2016;111(8):1589–1592. 10.1016/j.bpj.2016.08.044 27760345 PMC5071581

[43] Sekar RB, Periasamy A. Fluorescence resonance energy transfer (FRET) microscopy imaging of live cell protein localizations. The Journal of cell biology. 2003;160(5):629–633. 10.1083/jcb.200210140 12615908 PMC2173363

[44] Hall A. Rho GTPases and the actin cytoskeleton. Science. 1998;279(5350):509–514. 10.1126/science.279.5350.509 9438836

[45] Hall A, Nobes CD. Rho GTPases: molecular switches that control the organization and dynamics of the actin cytoskeleton. Philosophical Transactions of the Royal Society of London Series B: Biological Sciences. 2000;355(1399):965–970. 10.1098/rstb.2000.0632 11128990 PMC1692798

[46] Ridley AJ. Rho GTPases and cell migration. Journal of cell science. 2001;114(15):2713–2722. 11683406 10.1242/jcs.114.15.2713

[47] Khatibi S, Rios KI, Nguyen LK. Computational Modeling of the Dynamics of Spatiotemporal Rho GTPase Signaling: A Systematic Review In: Rho GTPases. Springer; 2018 p. 3–20. 10.1007/978-1-4939-8612-5_1 30062401

[48] Kholodenko BN. Cell-signalling dynamics in time and space. Nature reviews Molecular cell biology. 2006;7(3):165–176. 10.1038/nrm1838 16482094 PMC1679905

[49] Tyson JJ, Chen KC, Novak B. Sniffers, buzzers, toggles and blinkers: dynamics of regulatory and signaling pathways in the cell. Current opinion in cell biology. 2003;15(2):221–231. 10.1016/S0955-0674(03)00017-6 12648679

[50] Sailem H, Bousgouni V, Cooper S, Bakal C. Cross-talk between Rho and Rac GTPases drives deterministic exploration of cellular shape space and morphological heterogeneity. Open biology. 2014;4(1):130132 10.1098/rsob.130132 24451547 PMC3909273

[51] Byrne KM, Monsefi N, Dawson JC, Degasperi A, Bukowski-Wills JC, Volinsky N, et al Bistability in the Rac1, PAK, and RhoA signaling network drives actin cytoskeleton dynamics and cell motility switches. Cell systems. 2016;2(1):38–48. 10.1016/j.cels.2016.01.003 27136688 PMC4802415

[52] Marée AF, Jilkine A, Dawes A, Grieneisen VA, Edelstein-Keshet L. Polarization and movement of keratocytes: a multiscale modelling approach. Bulletin of mathematical biology. 2006;68(5):1169–1211. 10.1007/s11538-006-9131-7 16794915

[53] Devreotes PN, Bhattacharya S, Edwards M, Iglesias PA, Lampert T, Miao Y. Excitable signal transduction networks in directed cell migration. Annual review of cell and developmental biology. 2017;33:103–125. 10.1146/annurev-cellbio-100616-060739 PMC5792054 28793794

[54] Otsuji M, Ishihara S, Co C, Kaibuchi K, Mochizuki A, Kuroda S. A mass conserved reaction–diffusion system captures properties of cell polarity. PLoS computational biology. 2007;3(6). 10.1371/journal.pcbi.0030108 17559299 PMC1892603

[55] Jilkine A, Marée AF, Edelstein-Keshet L. Mathematical model for spatial segregation of the Rho-family GTPases based on inhibitory crosstalk. Bulletin of mathematical biology. 2007;69(6):1943–1978. 10.1007/s11538-007-9200-6 17457653

[56] Sakumura Y, Tsukada Y, Yamamoto N, Ishii S. A molecular model for axon guidance based on cross talk between rho GTPases. Biophysical journal. 2005;89(2):812–822. 10.1529/biophysj.104.055624 15923236 PMC1366631

[57] Falkenberg CV, Loew LM. Computational analysis of Rho GTPase cycling. PLoS computational biology. 2013;9(1). 10.1371/journal.pcbi.1002831 23326220 PMC3542069

[58] Hetmanski JH, Zindy E, Schwartz JM, Caswell PT. A MAPK-driven feedback loop suppresses Rac activity to promote RhoA-driven cancer cell invasion. PLoS computational biology. 2016;12(5). 10.1371/journal.pcbi.1004909 27138333 PMC4854413

[59] Letort G, Montagud A, Stoll G, Heiland R, Barillot E, Macklin P, et al PhysiBoSS: a multi-scale agent-based modelling framework integrating physical dimension and cell signalling. Bioinformatics. 2018;35(7):1188–1196. 10.1093/bioinformatics/bty766 PMC6449758 30169736

[60] Holmes WR, Edelstein-Keshet L. Analysis of a minimal Rho-GTPase circuit regulating cell shape. Physical Biology. 2016;13(4):046001 10.1088/1478-3975/13/4/046001 27434017

[61] Mori Y, Jilkine A, Edelstein-Keshet L. Wave-pinning and cell polarity from a bistable reaction-diffusion system. Biophysical journal. 2008;94(9):3684–3697. 10.1529/biophysj.107.120824 18212014 PMC2292363

[62] Vanderlei B, Feng JJ, Edelstein-Keshet L. A computational model of cell polarization and motility coupling mechanics and biochemistry. Multiscale Modeling & Simulation. 2011;9(4):1420–1443. 10.1137/100815335 22904684 PMC3419594

[63] Cusseddu D, Edelstein-Keshet L, Mackenzie JA, Portet S, Madzvamuse A. A coupled bulk-surface model for cell polarisation. Journal of theoretical biology. 2019;481:119–135. 10.1016/j.jtbi.2018.09.008 30205095

[64] Park J, Holmes WR, Lee SH, Kim HN, Kim DH, Kwak MK, et al Mechanochemical feedback underlies coexistence of qualitatively distinct cell polarity patterns within diverse cell populations. Proceedings of the National Academy of Sciences. 2017;114(28):E5750–E5759. 10.1073/pnas.1700054114 28655842 PMC5514712

[65] Holmes WR, Park J, Levchenko A, Edelstein-Keshet L. A mathematical model coupling polarity signaling to cell adhesion explains diverse cell migration patterns. PLoS computational biology. 2017;13(5):e1005524 10.1371/journal.pcbi.1005524 28472054 PMC5436877

[66] Mori Y, Jilkine A, Edelstein-Keshet L. Asymptotic and bifurcation analysis of wave-pinning in a reaction-diffusion model for cell polarization. SIAM journal on applied mathematics. 2011;71(4):1401–1427. 10.1137/10079118X 22171122 PMC3235655

[67] Cusseddu D, Edelstein-Keshet L, Mackenzie JA, Portet S, Madzvamuse A. A coupled bulk-surface model for cell polarisation. Journal of theoretical biology. 2019;481:119–135. 10.1016/j.jtbi.2018.09.008 30205095

[68] Wu T, Feng JJ. Modeling the mechanosensitivity of neutrophils passing through a narrow channel. Biophysical journal. 2015;109(11):2235–2245. 10.1016/j.bpj.2015.10.032 26636935 PMC4675883

[69] Diegmiller R, Montanelli H, Muratov CB, Shvartsman SY. Spherical caps in cell polarization. Biophysical journal. 2018;115(1):26–30. 10.1016/j.bpj.2018.05.033 29933887 PMC6035301

[70] Lin B, Holmes WR, Wang CJ, Ueno T, Harwell A, Edelstein-Keshet L, et al Synthetic spatially graded Rac activation drives cell polarization and movement. Proceedings of the National Academy of Sciences. 2012;109(52):E3668–E3677. 10.1073/pnas.1210295109 23185021 PMC3535611

[71] Camley BA, Zhao Y, Li B, Levine H, Rappel WJ. Periodic migration in a physical model of cells on micropatterns. Physical review letters. 2013;111(15):158102 10.1103/PhysRevLett.111.158102 24160631 PMC3855234

[72] Kopfer KH, Jäger W, Matthäus F. A mechanochemical model for rho GTPase mediated cell polarization. Journal of Theoretical Biology. 2020;504:110386 10.1016/j.jtbi.2020.110386 32653321

[73] Masuzzo P, Van Troys M, Ampe C, Martens L. Taking aim at moving targets in computational cell migration. Trends in cell biology. 2016;26(2):88–110. 10.1016/j.tcb.2015.09.003 26481052

[74] Lin B, Yin T, Wu YI, Inoue T, Levchenko A. Interplay between chemotaxis and contact inhibition of locomotion determines exploratory cell migration. Nature communications. 2015;6:6619 10.1038/ncomms7619 PMC4391292 25851023

[75] MacKay JL, Kumar S. Simultaneous and independent tuning of RhoA and Rac1 activity with orthogonally inducible promoters. Integrative Biology. 2014;6(9):885–894. 10.1039/c4ib00099d 25044255 PMC4141552

[76] Zmurchok C, Holmes WR. Simple Rho GTPase Dynamics Generate a Complex Regulatory Landscape Associated with Cell Shape. Biophysical Journal. 2020;118:1438–1454. 10.1016/j.bpj.2020.01.035 32084329 PMC7091515

[77] Mosier JA, Rahman-Zaman A, Zanotelli MR, VanderBurgh JA, Bordeleau F, Hoffman BD, et al Extent of cell confinement in microtracks affects speed and results in differential matrix strains. Biophysical Journal. 2019;117(9):1692–1701. 10.1016/j.bpj.2019.09.024 31623884 PMC6838744

[78] Houk AR, Jilkine A, Mejean CO, Boltyanskiy R, Dufresne ER, Angenent SB, et al Membrane tension maintains cell polarity by confining signals to the leading edge during neutrophil migration. Cell. 2012;148(1-2):175–188. 10.1016/j.cell.2011.10.050 22265410 PMC3308728

[79] Ohashi K, Fujiwara S, Mizuno K. Roles of the cytoskeleton, cell adhesion and rho signalling in mechanosensing and mechanotransduction. The Journal of Biochemistry. 2017;161(3):245–254. 28082721 10.1093/jb/mvw082

[80] Warner H, Wilson BJ, Caswell PT. Control of adhesion and protrusion in cell migration by Rho GTPases. Current opinion in cell biology. 2019;56:64–70. 10.1016/j.ceb.2018.09.003 30292078 PMC6368645

[81] Chagnon-Lessard S, Jean-Ruel H, Godin M, Pelling AE. Cellular orientation is guided by strain gradients. Integrative Biology. 2017;9(7):607–618. 10.1039/C7IB00019G 28534911

[82] Topaz CM, Ziegelmeier L, Halverson T. Topological data analysis of biological aggregation models. PloS one. 2015;10(5):e0126383 10.1371/journal.pone.0126383 25970184 PMC4430537

[83] Ulmer M, Ziegelmeier L, Topaz CM. A topological approach to selecting models of biological experiments. PloS one. 2019;14(3):e0213679 10.1371/journal.pone.0213679 30875410 PMC6420156

[84] Bhaskar D, Manhart A, Milzman J, Nardini JT, Storey KM, Topaz CM, et al Analyzing collective motion with machine learning and topology. Chaos: An Interdisciplinary Journal of Nonlinear Science. 2019;29(12):123125 10.1063/1.5125493 31893635 PMC7027427

[85] McGuirl MR, Volkening A, Sandstede B. Topological data analysis of zebrafish patterns. Proceedings of the National Academy of Sciences. 2020;117(10):5113–5124. 10.1073/pnas.1917763117 PMC7071871 32098851

[86] De Pascalis C, Etienne-Manneville S. Single and collective cell migration: the mechanics of adhesions. Molecular biology of the cell. 2017;28(14):1833–1846. 10.1091/mbc.e17-03-0134 28684609 PMC5541834

[87] Schumacher LJ, Kulesa PM, McLennan R, Baker RE, Maini PK. Multidisciplinary approaches to understanding collective cell migration in developmental biology. Open biology. 2016;6(6):160056 10.1098/rsob.160056 27278647 PMC4929938

[88] Kulawiak DA, Camley BA, Rappel WJ. Modeling contact inhibition of locomotion of colliding cells migrating on micropatterned substrates. PLoS computational biology. 2016;12(12):e1005239 10.1371/journal.pcbi.1005239 27984579 PMC5161303

[89] McLennan R, Dyson L, Prather KW, Morrison JA, Baker RE, Maini PK, et al Multiscale mechanisms of cell migration during development: theory and experiment. Development. 2012;139(16):2935–2944. 10.1242/dev.081471 22764050 PMC3403103

[90] McLennan R, Schumacher LJ, Morrison JA, Teddy JM, Ridenour DA, Box AC, et al Neural crest migration is driven by a few trailblazer cells with a unique molecular signature narrowly confined to the invasive front. Development. 2015;142(11):2014–2025. 10.1242/dev.117507 25977364

[91] Camley BA, Zhang Y, Zhao Y, Li B, Ben-Jacob E, Levine H, et al Polarity mechanisms such as contact inhibition of locomotion regulate persistent rotational motion of mammalian cells on micropatterns. Proceedings of the National Academy of Sciences. 2014;111(41):14770–14775. 10.1073/pnas.1414498111 25258412 PMC4205601

[92] Kulesa PM, Bailey CM, Kasemeier-Kulesa JC, McLennan R. Cranial neural crest migration: new rules for an old road. Developmental biology. 2010;344(2):543–554. 10.1016/j.ydbio.2010.04.010 20399765 PMC2914193

[93] Woods ML, Carmona-Fontaine C, Barnes CP, Couzin ID, Mayor R, Page KM. Directional collective cell migration emerges as a property of cell interactions. PLoS One. 2014;9(9). 10.1371/journal.pone.0104969 25181349 PMC4152153

[94] Méhes E, Vicsek T. Collective motion of cells: from experiments to models. Integrative biology. 2014;6(9):831–854. 10.1039/C4IB00115J 25056221

[95] Camley BA, Rappel WJ. Physical models of collective cell motility: from cell to tissue. Journal of physics D: Applied physics. 2017;50(11):113002 10.1088/1361-6463/aa56fe 28989187 PMC5625300

[96] Desai RA, Gopal SB, Chen S, Chen CS. Contact inhibition of locomotion probabilities drive solitary versus collective cell migration. Journal of The Royal Society Interface. 2013;10(88):20130717 10.1098/rsif.2013.0717 PMC3785843 24047876

[97] Hoehme S, Drasdo D. A cell-based simulation software for multi-cellular systems. Bioinformatics. 2010;26(20):2641–2642. 10.1093/bioinformatics/btq437 20709692 PMC2951083

[98] Mogilner A, Edelstein-Keshet L. A non-local model for a swarm. Journal of mathematical biology. 1999;38(6):534–570. 10.1007/s002850050158

[99] Volkening A, Sandstede B. Modelling stripe formation in zebrafish: an agent-based approach. Journal of the Royal Society Interface. 2015;12(112):20150812 10.1098/rsif.2015.0812 PMC4685853 26538560

[100] Cai D, Dai W, Prasad M, Luo J, Gov NS, Montell DJ. Modeling and analysis of collective cell migration in an in vivo three-dimensional environment. Proceedings of the National Academy of Sciences. 2016;113(15):E2134–E2141. 10.1073/pnas.1522656113 PMC4839456 27035964

[101] Stonko DP, Manning L, Starz-Gaiano M, Peercy BE. A mathematical model of collective cell migration in a three-dimensional, heterogeneous environment. PloS one. 2015;10(4). 10.1371/journal.pone.0122799 25875645 PMC4395426

[102] Shao D, Rappel WJ, Levine H. Computational model for cell morphodynamics. Physical review letters. 2010;105(10):108104 10.1103/PhysRevLett.105.108104 20867552 PMC3048783

[103] Camley BA. Collective gradient sensing and chemotaxis: modeling and recent developments. Journal of Physics: Condensed Matter. 2018;30(22):223001. 29644981 10.1088/1361-648X/aabd9f PMC6252055

[104] Merchant B, Feng JJ. A Rho-GTPase based model explains group advantage in collective chemotaxis of neural crest cells. Physical Biology. 2020;17(3):036002 10.1088/1478-3975/ab71f1 32000150

[105] Jain S, Cachoux VM, Narayana GH, de Beco S, D’Alessandro J, Cellerin V, et al The role of single-cell mechanical behaviour and polarity in driving collective cell migration. Nature Physics. 2020; p. 1–8. 10.1038/s41567-020-0875-z 32641972 PMC7343533

[106] Lo CM, Wang HB, Dembo M, Wang Yl. Cell movement is guided by the rigidity of the substrate. Biophysical journal. 2000;79(1):144–152. 10.1016/S0006-3495(00)76279-5 10866943 PMC1300921

[107] Schlüter DK, Ramis-Conde I, Chaplain MA. Computational modeling of single-cell migration: the leading role of extracellular matrix fibers. Biophysical journal. 2012;103(6):1141–1151. 10.1016/j.bpj.2012.07.048 22995486 PMC3446673

[108] Carmona-Fontaine C, Matthews HK, Kuriyama S, Moreno M, Dunn GA, Parsons M, et al Contact inhibition of locomotion in vivo controls neural crest directional migration. Nature. 2008;456(7224):957 10.1038/nature07441 19078960 PMC2635562

[109] Astin JW, Batson J, Kadir S, Charlet J, Persad RA, Gillatt D, et al Competition amongst Eph receptors regulates contact inhibition of locomotion and invasiveness in prostate cancer cells. Nature cell biology. 2010;12(12):1194 10.1038/ncb2122 21076414

[110] Roycroft A, Mayor R. Forcing contact inhibition of locomotion. Trends in cell biology. 2015;25(7):373–375. 10.1016/j.tcb.2015.05.001 25981318 PMC4509518

[111] Davis JR, Luchici A, Mosis F, Thackery J, Salazar JA, Mao Y, et al Inter-cellular forces orchestrate contact inhibition of locomotion. Cell. 2015;161(2):361–373. 10.1016/j.cell.2015.02.015 25799385 PMC4398973

[112] Lessey EC, Guilluy C, Burridge K. From mechanical force to RhoA activation. Biochemistry. 2012;51(38):7420–7432. 10.1021/bi300758e 22931484 PMC3567302

[113] Paksa A, Bandemer J, Hoeckendorf B, Razin N, Tarbashevich K, Minina S, et al Repulsive cues combined with physical barriers and cell-cell adhesion determine progenitor cell positioning during organogenesis. Nature communications. 2016;7:1–14. 10.1038/ncomms11288 27088892 PMC4837475

[114] Theveneau E, Steventon B, Scarpa E, Garcia S, Trepat X, Streit A, et al Chase-and-run between adjacent cell populations promotes directional collective migration. Nature cell biology. 2013;15(7):763 10.1038/ncb2772 23770678 PMC4910871

[115] Ng MR, Besser A, Brugge JS, Danuser G. Correction: Mapping the dynamics of force transduction at cell–cell junctions of epithelial clusters. eLife. 2015;4:e06656 10.7554/eLife.06656 25642646 PMC4311494

[116] Heinrich MA, Alert R, LaChance JM, Zajdel TJ, Košmrlj A, Cohen DJ. Size-dependent patterns of cell proliferation and migration in freely-expanding epithelia. eLife. 2020;9:e58945 10.7554/eLife.58945 32812871 PMC7498264

[117] Capuana L, Boström A, Etienne-Manneville S. Multicellular scale front-to-rear polarity in collective migration. Current opinion in cell biology. 2020;62:114–122. 10.1016/j.ceb.2019.10.001 31756576

[118] Manhart A, Windner S, Baylies M, Mogilner A. Mechanical positioning of multiple nuclei in muscle cells. PLoS computational biology. 2018;14(6):e1006208 10.1371/journal.pcbi.1006208 29889846 PMC6013246

[119] Odell GM, Oster G, Alberch P, Burnside B. The mechanical basis of morphogenesis: I. Epithelial folding and invagination. Developmental biology. 1981;85(2):446–462. 10.1016/0012-1606(81)90276-1 7196351

[120] Scarpa E, Mayor R. Collective cell migration in development. Journal of Cell Biology. 2016;212(2):143–155. 10.1083/jcb.201508047 PMC4738384 26783298

[121] Friedl P, Gilmour D. Collective cell migration in morphogenesis, regeneration and cancer. Nature reviews Molecular cell biology. 2009;10(7):445 10.1038/nrm2720 19546857

[122] Haeger A, Wolf K, Zegers MM, Friedl P. Collective cell migration: guidance principles and hierarchies. Trends in cell biology. 2015;25(9):556–566. 10.1016/j.tcb.2015.06.003 26137890

[123] Wu YI, Frey D, Lungu OI, Jaehrig A, Schlichting I, Kuhlman B, et al A genetically encoded photoactivatable Rac controls the motility of living cells. Nature. 2009;461(7260):104 10.1038/nature08241 19693014 PMC2766670

[124] O’Neill PR, Kalyanaraman V, Gautam N. Subcellular optogenetic activation of Cdc42 controls local and distal signaling to drive immune cell migration. Molecular biology of the cell. 2016;27(9):1442–1450. 10.1091/mbc.E15-12-0832 26941336 PMC4850032

[125] O’Neill PR, Castillo-Badillo JA, Meshik X, Kalyanaraman V, Melgarejo K, Gautam N. Membrane flow drives an adhesion-independent amoeboid cell migration mode. Developmental cell. 2018;46(1):9–22. 10.1016/j.devcel.2018.05.029 29937389 PMC6048972

[126] Meier B, Zielinski A, Weber C, Arcizet D, Youssef S, Franosch T, et al Chemotactic cell trapping in controlled alternating gradient fields. Proceedings of the National Academy of Sciences. 2011;108(28):11417–11422. 10.1073/pnas.1014853108 21709255 PMC3136296

[127] Lockley R, Ladds G, Bretschneider T. Image based validation of dynamical models for cell reorientation. Cytometry Part A. 2015;87(6):471–480. 10.1002/cyto.a.22600 PMC4890678 25492625

[128] Bretscher A, Edwards K, Fehon RG. ERM proteins and merlin: integrators at the cell cortex. Nature reviews Molecular cell biology. 2002;3(8):586–599. 10.1038/nrm882 12154370

[129] Das T, Safferling K, Rausch S, Grabe N, Boehm H, Spatz JP. A molecular mechanotransduction pathway regulates collective migration of epithelial cells. Nature cell biology. 2015;17(3):276 10.1038/ncb3115 25706233

[130] Londono C, Loureiro MJ, Slater B, Lücker PB, Soleas J, Sathananthan S, et al Nonautonomous contact guidance signaling during collective cell migration. Proceedings of the National Academy of Sciences. 2014;111(5):1807–1812. 10.1073/pnas.1321852111 24449852 PMC3918762

[131] Reffay M, Parrini MC, Cochet-Escartin O, Ladoux B, Buguin A, Coscoy S, et al Interplay of RhoA and mechanical forces in collective cell migration driven by leader cells. Nature cell biology. 2014;16(3):217 10.1038/ncb2917 24561621

[132] Vedula SRK, Leong MC, Lai TL, Hersen P, Kabla AJ, Lim CT, et al Emerging modes of collective cell migration induced by geometrical constraints. Proceedings of the National Academy of Sciences. 2012;109(32):12974–12979. 10.1073/pnas.1119313109 22814373 PMC3420172

[133] Peyret G, Mueller R, d’Alessandro J, Begnaud S, Marcq P, Mège RM, et al Sustained oscillations of epithelial cell sheets. Biophysical journal. 2019;117(3):464–478. 10.1016/j.bpj.2019.06.013 31307676 PMC6697349

[134] Zaritsky A, Welf ES, Tseng YY, Rabadán MA, Serra-Picamal X, Trepat X, et al Seeds of locally aligned motion and stress coordinate a collective cell migration. Biophysical journal. 2015;109(12):2492–2500. 10.1016/j.bpj.2015.11.001 26682808 PMC4699880

[135] Zaritsky A, Tseng YY, Rabadán MA, Krishna S, Overholtzer M, Danuser G, et al Diverse roles of guanine nucleotide exchange factors in regulating collective cell migration. J Cell Biol. 2017;216(6):1543–1556. 10.1083/jcb.201609095 28512143 PMC5461017

[136] Shi Z, Graber ZT, Baumgart T, Stone HA, Cohen AE. Cell membranes resist flow. Cell. 2018;175(7):1769–1779. 10.1016/j.cell.2018.09.054 30392960 PMC6541487

[137] Kasemeier-Kulesa JC, Morrison JA, Lefcort F, Kulesa PM. TrkB/BDNF signalling patterns the sympathetic nervous system. Nature communications. 2015;6:8281 10.1038/ncomms9281 PMC4586040 26404565

[138] Van Liedekerke P, Palm M, Jagiella N, Drasdo D. Simulating tissue mechanics with agent-based models: concepts, perspectives and some novel results. Computational particle mechanics. 2015;2(4):401–444. 10.1007/s40571-015-0082-3

[139] Van Liedekerke P, Buttenschön A, Drasdo D. Off-lattice agent-based models for cell and tumor growth: numerical methods, implementation, and applications In: Numerical Methods and Advanced Simulation in Biomechanics and Biological Processes. Elsevier; 2018 p. 245–267.

[140] Yang Y, Jolly MK, Levine H. Computational Modeling of Collective Cell Migration: Mechanical and Biochemical Aspects In: Cell Migrations: Causes and Functions. Springer; 2019 p. 1–11. 10.1007/978-3-030-17593-1_1 31612450

[141] Fletcher AG, Osborne JM, Maini PK, Gavaghan DJ. Implementing vertex dynamics models of cell populations in biology within a consistent computational framework. Progress in biophysics and molecular biology. 2013;113(2):299–326. 10.1016/j.pbiomolbio.2013.09.003 24120733

[142] Osborne JM. Multiscale model of colorectal cancer using the cellular Potts framework. Cancer informatics. 2015;14:CIN–S19332. 10.4137/CIN.S19332 PMC4598229 26461973

[143] Osborne JM, Fletcher AG, Pitt-Francis JM, Maini PK, Gavaghan DJ. Comparing individual-based approaches to modelling the self-organization of multicellular tissues. PLoS computational biology. 2017;13(2):e1005387 10.1371/journal.pcbi.1005387 28192427 PMC5330541

[144] Swat MH, Thomas GL, Belmonte JM, Shirinifard A, Hmeljak D, Glazier JA. Multi-scale modeling of tissues using CompuCell3D In: Methods in cell biology. vol. 110 Elsevier; 2012 p. 325–366. 22482955 10.1016/B978-0-12-388403-9.00013-8 PMC3612985

[145] Starruß J, de Back W, Brusch L, Deutsch A. Morpheus: a user-friendly modeling environment for multiscale and multicellular systems biology. Bioinformatics. 2014;30(9):1331–1332. 10.1093/bioinformatics/btt772 24443380 PMC3998129

[146] Mulberry N, Edelstein-Keshet L. Self-organized multicellular structures from simple cell signaling: a computational model. Physical Biology. 2020;. 10.1088/1478-3975/abb2dc 33210618

[147] Bui J, Conway DE, Heise RL, Weinberg SH. Mechanochemical coupling and junctional forces during collective cell migration. Biophysical journal. 2019;117(1):170–183. 10.1016/j.bpj.2019.05.020 31200935 PMC6626874

[148] Jamali Y, Azimi M, Mofrad MR. A sub-cellular viscoelastic model for cell population mechanics. PLoS One. 2010;5(8):e12097 10.1371/journal.pone.0012097 20856895 PMC2938372

[149] Osterfield M, Du X, Schüpbach T, Wieschaus E, Shvartsman SY. Three-dimensional epithelial morphogenesis in the developing Drosophila egg. Developmental cell. 2013;24(4):400–410. 10.1016/j.devcel.2013.01.017 23449472 PMC4080892

[150] Aracena J, González M, Zuñiga A, Mendez MA, Cambiazo V. Regulatory network for cell shape changes during Drosophila ventral furrow formation. Journal of Theoretical Biology. 2006;239(1):49–62. 10.1016/j.jtbi.2005.07.011 16139845

[151] Tarle V, Gauquelin E, Vedula S, D’Alessandro J, Lim C, Ladoux B, et al Modeling collective cell migration in geometric confinement. Physical biology. 2017;14(3):035001 10.1088/1478-3975/aa6591 28467320

[152] Albert PJ, Schwarz US. Dynamics of cell ensembles on adhesive micropatterns: bridging the gap between single cell spreading and collective cell migration. PLoS computational biology. 2016;12(4). 10.1371/journal.pcbi.1004863 27054883 PMC4824460

[153] Schnyder SK, Molina JJ, Tanaka Y, Yamamoto R. Collective motion of cells crawling on a substrate: roles of cell shape and contact inhibition. Scientific reports. 2017;7(1):1–14. 28701766 10.1038/s41598-017-05321-0 PMC5507894

[154] Drasdo D, Kree R, McCaskill J. Monte Carlo approach to tissue-cell populations. Physical review E. 1995;52(6):6635 10.1103/PhysRevE.52.6635 9964180

[155] Palsson E, Othmer HG. A model for individual and collective cell movement in Dictyostelium discoideum. Proceedings of the National Academy of Sciences. 2000;97(19):10448–10453. 10.1073/pnas.97.19.10448 PMC27044 10984537

[156] Palsson E. A 3-D model used to explore how cell adhesion and stiffness affect cell sorting and movement in multicellular systems. Journal of Theoretical Biology. 2008;254(1):1–13. 18582903 10.1016/j.jtbi.2008.05.004

[157] Frascoli F, Hughes BD, Zaman MH, Landman KA. A computational model for collective cellular motion in three dimensions: general framework and case study for cell pair dynamics. PloS one. 2013;8(3). 10.1371/journal.pone.0059249 23527148 PMC3602115

[158] Lin SZ, Ye S, Xu GK, Li B, Feng XQ. Dynamic migration modes of collective cells. Biophysical journal. 2018;115(9):1826–1835. 10.1016/j.bpj.2018.09.010 30297134 PMC6224637

[159] Lin SZ, Bi D, Li B, Feng XQ. Dynamic instability and migration modes of collective cells in channels. Journal of the Royal Society Interface. 2019;16(156):20190258 10.1098/rsif.2019.0258 PMC6685016 31362619

[160] Doxzen K, Vedula SRK, Leong MC, Hirata H, Gov NS, Kabla AJ, et al Guidance of collective cell migration by substrate geometry. Integrative biology. 2013;5(8):1026–1035. 10.1039/c3ib40054a 23784144

[161] Winkler B, Aranson IS, Ziebert F. Confinement and substrate topography control cell migration in a 3D computational model. Communications Physics. 2019;2(1):1–11.

[162] Hannezo E, Prost J, Joanny JF. Theory of epithelial sheet morphology in three dimensions. Proceedings of the National Academy of Sciences. 2014;111(1):27–32. 10.1073/pnas.1312076111 PMC3890844 24367079

[163] Banerjee S, Marchetti MC. Continuum models of collective cell migration In: Cell Migrations: Causes and Functions. Springer; 2019 p. 45–66. 10.1007/978-3-030-17593-1_4 31612453

[164] Arciero JC, Mi Q, Branca MF, Hackam DJ, Swigon D. Continuum model of collective cell migration in wound healing and colony expansion. Biophysical journal. 2011;100(3):535–543. 10.1016/j.bpj.2010.11.083 21281567 PMC3030184

[165] Escribano J, Sunyer R, Sánchez MT, Trepat X, Roca-Cusachs P, García-Aznar JM. A hybrid computational model for collective cell durotaxis. Biomechanics and modeling in mechanobiology. 2018;17(4):1037–1052. 10.1007/s10237-018-1010-2 29500553

[166] Degond P, Manhart A, Yu H. An age-structured continuum model for myxobacteria. Mathematical Models and Methods in Applied Sciences. 2018;28(09):1737–1770. 10.1142/S0218202518400043

[167] Aceves-Sanchez P, Degond P, Keaveny EE, Manhart A, Merino-Aceituno S, Peurichard D. Large-scale dynamics of self-propelled particles moving through obstacles: model derivation and pattern formation. arXiv preprint arXiv:200412638. 2020;. 10.1007/s11538-020-00805-z PMC7519010 32978682

[168] Yang Y, Levine H. Leader-cell-driven epithelial sheet fingering. Physical Biology. 2020;17(4):046003 10.1088/1478-3975/ab907e 32369794

[169] Van Aelst L, Symons M. Role of Rho family GTPases in epithelial morphogenesis. Genes & development. 2002;16(9):1032–1054. 10.1101/gad.978802 12000787

[170] Zegers MM, Friedl P. Rho GTPases in collective cell migration. Small GTPases. 2014;5(3):e983869 10.4161/sgtp.28997 PMC4114924 25054920

[171] Vishwakarma M, Di Russo J, Probst D, Schwarz US, Das T, Spatz JP. Mechanical interactions among followers determine the emergence of leaders in migrating epithelial cell collectives. Nature communications. 2018;9(1):3469 10.1038/s41467-018-05927-6 PMC6110746 30150695

[172] Wong IY, Javaid S, Wong EA, Perk S, Haber DA, Toner M, et al Collective and individual migration following the epithelial–mesenchymal transition. Nature materials. 2014;13(11):1063–1071. 10.1038/nmat4062 25129619 PMC4209198

[173] Park J, Kim DH, Shah SR, Kim HN, Kim P, Quiñones-Hinojosa A, et al Switch-like enhancement of epithelial-mesenchymal transition by YAP through feedback regulation of WT1 and Rho-family GTPases. Nature communications. 2019;10(1):1–15. 10.1038/s41467-019-10729-5 31243273 PMC6594963

[174] Roux C, Duperray A, Laurent VM, Michel R, Peschetola V, Verdier C, et al Prediction of traction forces of motile cells. Interface focus. 2016;6(5):20160042 10.1098/rsfs.2016.0042 27708765 PMC4992744

[175] Marzban B. A multiphysics computational framework for understanding cell and microtissue morphogenesis [PhD Thesis]. University of Rhode Island; 2018. Available from: 10.23860/diss-marzban-bahador-2018.

[176] Satulovsky J, Lui R, Wang Yl. Exploring the control circuit of cell migration by mathematical modeling. Biophysical journal. 2008;94(9):3671–3683. 10.1529/biophysj.107.117002 18199677 PMC2292371

[177] Savill NJ, Hogeweg P. Modelling morphogenesis: from single cells to crawling slugs. Journal of theoretical biology. 1997;184(3):229–235. 10.1006/jtbi.1996.0237 31940735

[178] Marée AF, Hogeweg P. How amoeboids self-organize into a fruiting body: multicellular coordination in Dictyostelium discoideum. Proceedings of the National Academy of Sciences. 2001;98(7):3879–3883. 10.1073/pnas.061535198 PMC31146 11274408

[179] Hogeweg P. Evolving Mechanisms of Morphogenesis: on the Interplay between Differential Adhesionand Cell Differentiation. J theor Biol. 2000;203:317–333. 10.1006/jtbi.2000.1087 10736211

[180] Vroomans RM, Hogeweg P, ten Tusscher KH. Segment-specific adhesion as a driver of convergent extension. PLoS computational biology. 2015;11(2):e1004092 10.1371/journal.pcbi.1004092 25706823 PMC4338282

[181] Rubinacci S, Graudenzi A, Caravagna G, Mauri G, Osborne J, Pitt-Francis J, et al Cognac: a chaste plugin for the multiscale simulation of gene regulatory networks driving the spatial dynamics of tissues and cancer. Cancer informatics. 2015;14:CIN–S19965. 10.4137/CIN.S19965 26380549 PMC4559197

[182] Toda S, Blauch LR, Tang SK, Morsut L, Lim WA. Programming self-organizing multicellular structures with synthetic cell-cell signaling. Science. 2018;361(6398):156–162. 29853554 10.1126/science.aat0271 PMC6492944

[183] Lam C, Morsut L. A Modular Computational Framework for the Design of Multicellular Genetic Circuits of Morphogenesis. bioRxiv. 2020; p. 784496.

[184] Chauhan BK, Lou M, Zheng Y, Lang RA. Balanced Rac1 and RhoA activities regulate cell shape and drive invagination morphogenesis in epithelia. Proceedings of the National Academy of Sciences. 2011;108(45):18289–18294. 10.1073/pnas.1108993108 PMC3215052 22021442

[185] Rejniak KA, Anderson AR. Hybrid models of tumor growth. Wiley Interdisciplinary Reviews: Systems Biology and Medicine. 2011;3(1):115–125. 21064037 10.1002/wsbm.102 PMC3057876

[186] Magno R, Grieneisen VA, Marée AF. The biophysical nature of cells: potential cell behaviours revealed by analytical and computational studies of cell surface mechanics. BMC biophysics. 2015;8(1):8 10.1186/s13628-015-0022-x 26023328 PMC4446964

